# Efficient Approach to Color Image Segmentation Based on Multilevel Thresholding Using EMO Algorithm by Considering Spatial Contextual Information

**DOI:** 10.3390/jimaging9040074

**Published:** 2023-03-23

**Authors:** Srikanth Rangu, Rajagopal Veramalla, Surender Reddy Salkuti, Bikshalu Kalagadda

**Affiliations:** 1 Department of ECE, Kakatiya Institute of Technology and Science, Warangal 506015, India; 2 Department of Railroad and Electrical Engineering, Woosong University, Daejeon 34606, Republic of Korea; 3 Department of ECE, Kakatiya University, Warangal 506009, India

**Keywords:** color image, multilevel thresholding, Otsu’s and Kapur’s methods, spatial contextual information, inter-class variance, entropy

## Abstract

The process of image segmentation is partitioning an image into its constituent parts and is a significant approach for extracting interesting features from images. Over a couple of decades, many efficient image segmentation approaches have been formulated for various applications. Still, it is a challenging and complex issue, especially for color image segmentation. To moderate this difficulty, a novel multilevel thresholding approach is proposed in this paper based on the electromagnetism optimization (EMO) technique with an energy curve, named multilevel thresholding based on EMO and energy curve (MTEMOE). To compute the optimized threshold values, Otsu’s variance and Kapur’s entropy are deployed as fitness functions; both values should be maximized to locate optimal threshold values. In both Kapur’s and Otsu’s methods, the pixels of an image are classified into different classes based on the threshold level selected on the histogram. Optimal threshold levels give higher efficiency of segmentation; the EMO technique is used to find optimal thresholds in this research. The methods based on an image’s histograms do not possess the spatial contextual information for finding the optimal threshold levels. To abolish this deficiency an energy curve is used instead of the histogram and this curve can establish the spatial relationship of pixels with their neighbor pixels. To study the experimental results of the proposed scheme, several color benchmark images are considered at various threshold levels and compared with other meta-heuristic algorithms: multi-verse optimization, whale optimization algorithm, and so on. The investigational results are illustrated in terms of mean square error, peak signal-to-noise ratio, the mean value of fitness reach, feature similarity, structural similarity, variation of information, and probability rand index. The results reveal that the proposed MTEMOE approach overtops other state-of-the-art algorithms to solve engineering problems in various fields.

## 1. Introduction

Digital image segmentation is a technique of partitioning the image into regions to extract information about features of an image with homogeneous features in terms of intensity level, texture structure, color information, etc. The image segmentation schemes available from the literature, multi-level thresholding [1] of grayscale on the histogram of an image is a highly established method and is used in various applications from satellite image segmentation [2,3,4] to medical images. The important multilevel thresholding-based segmentation techniques are Kapur’s and Otsu’s methods [5,6]. Segmentation can often be used as a preprocessing step in object recognition, computer vision, image analysis, and so on in different applications such as medical [7], agricultural, industrial, fault detection, weather forecasting, etc. In general, the majority of segmentation techniques are based on discontinuity and similarity; among abundant methods available thresholding is the most important technique for both grayscale and color images.

Image segmentation is a significant step in image processing. Major advances in image segmentation are in the area of biomedical imaging to investigate the function, structure, and pathology of the human body, and in other industrial applications from robotics to satellite image segmentation.

In the multilevel thresholding method of segmentation, the pixels are grouped into different classes or groups (two or more) based on the gray-levels and multiple threshold values. The quality level of segmentation is affected by the technique used to compute threshold values. The use of a classical or traditional method of selecting the thresholds is computationally expensive as the technique needs to search in a huge range of sample space to identify the optimized levels using the objective function; at this stage, optimization techniques can be applicable and then there is a scope of research computing the optimized threshold levels.

The various significant multilevel thresholding approaches are based on image histograms. The techniques based on histograms have two major disadvantages, which are (i) spatial contextual information (relationships among the pixels in an image) not considered for finding the histogram, which leads to less efficiency in computing the optimized threshold levels on the histogram, and (ii) methods based on the histogram are incompetent for applications of segmentation with thresholding levels greater than two (MT).

Techniques with histogram plots are incapable of owning spatial contextual information to compute optimized thresholds. To conquer the drawbacks of the histogram of an image, a novel methodology is proposed: multilevel thresholding based on EMO and energy curve (MTEMOE). A curve that has similar characteristics to the histogram and the spatial contextual information of image pixels is named an “energy curve” and can be used in place of the histogram; an electro-magnetism optimization algorithm is used to select and optimize gray levels; an Energy Curve characteristics are similar to a histogram. For each value in an image, energy is computed in the grayscale range of that image. The threshold levels can be computed based on valleys and peak points on the energy curve.

In general, to find out the optimized threshold values, there are two types of computational techniques, called parametric and nonparametric [8]. In the case of parametric techniques, statistical parameters are used, depending on initial conditions, and hence are inflexible to be applied. In the case of nonparametric techniques, thresholds are computed based on some criteria such as Otsu’s inter-class variance and Kapurs’s entropy functions [9,10,11]. The thresholding method holds properties such as simplicity [12,13], accuracy, and robustness, which can be classified into two major categories: bi-level and multilevel [11]; the pixels of an image are classified into different classes based on the threshold level selected on the histogram. All the pixels are grouped into two classes based on threshold level in the case of bi-level thresholding. In the second category of multilevel thresholding, pixels are categorized into more than two classes. Nevertheless, the primary constraints in multilevel thresholding are accuracy, stability, time for execution, and so on.

In the case of color images [14], each pixel consists of three components (red, green, and blue) [15]; due to this heavy load, the segmentation of color images might be more exigent and intricate. Accordingly, it is essential to find the optimal thresholds by using optimization algorithms by maximizing the inter-class variance in Otsu’s method and the histogram entropy in the case of Kapur’s method on a histogram of an image. As per the no-free-lunch (NFL) principle [16], no algorithm can solve all types of optimization problems [17]; one optimization algorithm may be very useful in one type of application and not succeed in solving other kinds of applications; thus, it is indispensable to devise and transform new algorithms.

Techniques with histogram plots are incapable of owning spatial contextual information to compute optimized thresholds. To conquer the drawbacks of the histogram of an image, a novel methodology is presented in this chapter; a curve that has similar characteristics to that of the histogram and considers spatial contextual information of image pixels named an “energy curve” [6] can be used in place of the histogram; the harmony search algorithm [5] is used to select optimized gray levels; energy curve characteristics are similar to a histogram. For each value in an image, energy is computed in the grayscale range of that image. The threshold levels can be computed based on valleys and peak points on the Energy Curve. In the literature, numerous optimization techniques along with the efficiencies and applications in particular fields are available, to mention a few, PSO [18], ACO [19], BFO [20], ABC [21], GWO [22], MFO [23], SSA [24], FA [25], WOA [26], SCA [27], KHO [28], BA [29], FPA [30], and MVO [31]. Moreover, several modified algorithms have been used in the multilevel thresholding field. For example, Chen et al. [32] proposed an improvised algorithm (IFA) to segment compared with PSO [33] and other methods [15,34].

From the above discussion, the techniques mentioned above mainly spotlight gray-scale images and extend to color images to some scale. Additionally, color satellite images have the features of complex backgrounds and poor resolution [35]; in this situation, it is very difficult to segment such color images. In this article, a new approach is projected for color image segmentation [36,37] and it aims at satellite images from experimental results. The proposed method is based on Kapur’s and Otsu’s methods with EMO on the energy curve to find optimal threshold levels. In a clearer way, the proposed model uses the energy curve instead of the histogram of an image. Multilevel thresholding [38,39] with EMO on energy curve, named MTEMOE, for color image segmentation, improves spotless performance in many aspects. The proposed segmentation approach is experienced on color images including satellite images and natural images and compared with competitive algorithms: MFO, WOA, FPA, MVO, SCA, ACO, ABC, and PSO. The segmented images are evaluated concerning seven metrics, which validate the dominance of MTEMOE.

## 2. Multilevel Thresholding

### 2.1. Otsu Method

This technique [5,9] is used for multi-level thresholding (MT), in which gray levels will be partitioned into different regions or classes; in this process thresholding (th) levels are selected; the set of rules to be followed for bi-level thresholding are

(1) C1←p if 0≤p<th,C2←p if th≤p<L−1

where C1 and C2 are two classes, p indicates the pixel value for the gray levels $\left\{1,2,3,\ldots ,L-1\right\}$ in an image and L−1 indicates the maximum gray level. If the gray level is below the threshold th then that pixel is grouped into class C1, else it is grouped into class C2. The set of rules for multi-level thresholding (MT) are

(2) C1←p if 0≤p<th1C2←p if th1≤p<th2Ci←p if thi≤p<thi+1Cn←p if thn≤p<thn+1

From Equation (2), C1,C2,…,Cn indicates different classes, and threshold levels to find objects represented by $\left\{th1,th2,...,thi,thi+1,thn\right\}$; these thresholds can be computed based on either a histogram or an energy curve. By use of these threshold levels, all the pixels will be classified into different classes or exclusive regions. The significant methods of segmentation of images based on threshold levels are Otsu’s and Kapur’s methods and, in both cases, threshold levels can be computed by maximizing the cost function (inter-class variance). In this work, optimized threshold levels are used by Otsu’s method th values [23]. In this method, inter-class variance is considered the objective function, also called a cost function. For experimentation, grayscale images are considered. The below expression gives the probability distribution for each gray level

(3) $${Ph}_{c}^{i}=\frac{h_{c}^{i}}{NP},\sum_{i=1}^{NP}{Ph}_{c}^{i}=1$$

From Equation (3), the pixel value is denoted by i, with the range of grayscale is (0≤i≤L−1), where c=1,2,3 for RGB and c=1 for a grayscale image, and total image pixels are represented by NP; the histogram of considered images is represented by $h_{c}^{i}$. In bi-level thresholding, the total pixels in the image are grouped into two classes

(4) $$C1=\frac{{Ph}_{1}^{c}}{w_{0}^{c}(th)},...\frac{{Ph}_{th}^{c}}{w_{0}^{c}(th)},C2=\frac{{Ph}_{th+1}^{c}}{w_{1}^{c}(th)},...\frac{{Ph}_{L}^{c}}{w_{1}^{c}(th)}$$

whereas $w_{0}\left(th\right)$ and $w_{1}\left(th\right)$ are the probabilities distributions for C1 and C2, as is shown below as

(5) $$w_{0}^{c}\left(th\right)=\sum_{j=1}^{th}{Ph}_{i}^{c}{,w}_{1}^{c}\left(th\right)=\sum_{j=th+1}^{th}{Ph}_{i}^{c}$$

The means of two classes $\mu_{0}^{c}$ and $\mu_{1}^{c}$ are computed by Equation (6), and the variance between classes $\sigma^{2^{c}}$ being given by Equation (7).

(6) $$\mu_{0}^{c}=\sum_{i=1}^{th}\frac{{iPh}_{i}^{c}}{w_{0}^{c}(th)},\mu_{1}^{c}=\sum_{i=th+1}^{L}\frac{{iPh}_{i}^{c}}{w_{1}^{c}(th)}$$

(7) $$\sigma^{2^{c}}=\sigma_{1}^{c}+\sigma_{2}^{c}$$

Notice that, for both Equations (6) and (7), c is determined by the type of image, where $\sigma_{1}^{c}$ and $\sigma_{2}^{c}$ in Equation (5) are the variances of classes C1 and C2 which are given in Equation (8).

(8) $$\sigma_{1}^{c}=w_{0}^{c}{\left(\mu_{0}^{c}+\mu_{T}^{c}\right)}^{2},\sigma_{2}^{c}=w_{1}^{c}{\left(\mu_{1}^{c}+\mu_{T}^{c}\right)}^{2}$$

where $\mu_{T}^{c}=w_{0}^{c}\mu_{0}^{c}+w_{1}^{c}\mu_{1}^{c}$ and $w_{0}^{c}+w_{1}^{c}=1.$ Based on the values $\sigma_{1}^{c}$ and $\sigma_{2}^{c}$, Equation (9) presents the objective function:

(9) $$J\left(th\right)=\max\left(\sigma^{2^{c}}\left(th\right)\right),0\leq th\leq L-1$$

From Equation (9), $\sigma^{2^{c}}\left(th\right)$ is the total variance between two various regions after segmentation by Otsu’s scheme [40,41] for given th; the optimization techniques required to find the threshold level (th) by maximizing the fitness function are as shown in Equation (8). Similarly for multi-level thresholding (MT), the objective (or fitness) function J(th), shown in Equation (11) to segment an image into k classes, requires k variances.

(10) $$J\left(TH\right)=\max\left(\sigma^{2^{c}}\left({th}_{i}\right)\right),0\leq {th}_{i}\leq L-1,\;where\;i=1,2\ldots ,k$$

where TH is a vector, $TH=\left[{th}_{1},{th}_{2},{th}_{3}\ldots \ldots {th}_{k-1}\right]$ for multi-level thresholding, and the variances between classes can be computed from Equation (12).

(11) $$\sigma^{2^{c}}=\sum_{i=1}^{k}\sigma_{i}^{c}=\sum_{i=1}^{k}w_{i}^{c}{\left(\mu_{i}^{c}-\mu_{T}^{c}\right)}^{2}$$

where i_th_ represents i class, $w_{i}^{c}$ indicates probability of $i_{th}$ classes and $\mu_{j}^{c}$ is the mean of the ith class. For MT segmentation, these parameters are anticipated as below:

(12) $$w_{0}^{c}\left(th\right)=\sum_{i=1}^{{th}_{1}}{Ph}_{i}^{c}{,w}_{1}^{c}\left(th\right)=\sum_{i={th}_{1}+1}^{{th}_{1}}{Ph}_{i}^{c}\cdots w_{k-1}^{c}\left(th\right)=\sum_{i={th}_{k}+1}^{{th}_{1}}{Ph}_{i}^{c}$$

Furthermore, the averages of each class can be computed as

(13) $$\mu_{0}^{c}=\sum_{i=1}^{{th}_{1}}\frac{{iPh}_{i}^{c}}{w_{0}^{c}({th}_{1})},\mu_{1}^{c}=\sum_{i={th}_{1}+1}^{{th}_{2}}\frac{{iPh}_{i}^{c}}{w_{0}^{c}({th}_{2})}\cdots \mu_{k-1}^{c}=\sum_{i={th}_{k}+1}^{L}\frac{{iPh}_{i}^{c}}{w_{1}^{c}({th}_{k})}$$

### 2.2. Multilevel Thresholding with Kapur’s Method

One more important nonparametric technique that is used to compute the optimal threshold values is Kapur’s method, entropy as an objective function. This method focuses on finding the optimal thresholds by maximizing the overall entropy. The entropy measures the compactness and separability between classes. For the multilevel, the objective function of Kapur’s method is defined as,

(14) $$J\left(TH\right)=\max\left(\sum_{i=1}^{k}H_{i}^{C}\right),0\leq {th}_{i}\leq L-1,\;where\;i=1,2\ldots k$$

where TH is a vector, $TH=\left[{th}_{1},{th}_{2},{th}_{3}\ldots \ldots {th}_{k-1}\right]$. Each entropy is calculated separately with its th value, given for k entropies

(15) $$H_{1}^{c}=\sum_{i=1}^{{th}_{1}}\frac{{Ph}_{i}^{c}}{w_{0}^{c}}\ln\left(\frac{{Ph}_{i}^{c}}{w_{0}^{c}}\right)H_{2}^{c}=\sum_{i=1}^{{th}_{1}}\frac{{Ph}_{i}^{c}}{w_{1}^{c}}\ln\left(\frac{{Ph}_{i}^{c}}{w_{1}^{c}}\right)\cdots H_{k}^{c}=\sum_{i={th}_{k}+1}^{{th}_{1}}\frac{{Ph}_{i}^{c}}{w_{k-1}^{c}}\ln\left(\frac{{Ph}_{i}^{c}}{w_{k-1}^{c}}\right)$$

${Ph}_{i}^{c}$ is the probability distribution of the particular intensity levels and it is obtained using (5). The values of the probability occurrence ($w_{0}^{c}$, $w_{1}^{c}$, $w_{2}^{c}$, …,$w_{k-1}^{c}$) of the 𝑘 classes are obtained using (12). In the end, by using Equation (2) classify the pixels into various classes.

### 2.3. Electro-Magnetism Optimization (EMO) Algorithm

The EMO [12] can be used to discover the solutions to global problems which are nonlinear in nature, and it can be used for minimization and maximization problems. For maximizing $\left(x\right),x=\left\{x_{1},x_{1},\ldots \;x_{1}\right\}\in R$ where x∈R, whereas $X=\left\{x\in R|l_{1}\leq x_{i}\leq u_{i},i=1,2,\ldots \;n\right\}$ is a solution set limited between ($l_{1}$) and ($u_{i}$) lower and upper limits, respectively. The EMO uses N, n-dimensional points $x_{i,t}$ as a population, the X indicates a solution set from the above expression, and t represents several generations or iterations by using the algorithm. Similar to other evolutionary optimization techniques, in EMO the initial population can also be taken as $S_{t}=\{x_{1,t},x_{2,t}\ldots ,x_{N,t}\}$ (being t = 1), selected from uniformly distributed random samples of the search region, X, whereas $S_{t}$ is the resultant solution set at the t^th^ iteration. At the first iteration $S_{t}$ should be initialized by arbitrary values randomly, then the EMO algorithm executes until the stopping criterion is satisfied.

In every iteration of EMO, two essential operations will take place; the first operation is the solution set $S_{t}$ moved to another different location or solution by means of the attraction and repulsion mechanism of the electromagnetism theory [11]; in the next operation positions moved as per the electromagnetism technique are auxiliary moved locally by local search and reach a member of $S_{t+1}$ in the (t + 1)th iteration. These two operations bring the solutions to the set close to global optimization solutions.

In EMO, similarly to electromagnetism theory, each solution $x_{i,t}\in S_{t}$ is treated as a charged particle, whereas the magnitude of the particle’s charge is treated as an object function, the solutions with better or optimal (higher/lower) object functions are associated with higher charges than the other set of solutions and also have a greater repulsion–attraction mechanism. In the evolution process of EMO, the points or solutions with higher charges can attract other points in the search space $S_{t}$ and points with a lower charge repel other points.

The total force $F_{i}^{t}$ exerted at each point, ($x_{i,t}),$ can be calculated by a combination of attraction-repulsion forces and each $x_{i,t}\in S_{t}$ is moved towards its total force to the location $y_{i,t}$. After this step, a local search algorithm is used to find the vicinity of every $y_{i,t}$ by $y_{i,t}$ to $z_{i,t}$. The solution set $x_{i,t+1}\in S_{t+1}$ at (t + 1)th iteration is subsequently computed as:

(16) $$x_{i,t+1}=y_{i,t}\;if\;f\left(y_{i,t}\right)\leq f(z_{i,t})\;x_{i,t+1}=z_{i,t},\;otherwise$$

A detailed description of each step in EMO is given in Algorithm 1 below.
**Algorithm 1:** A summary of the EMO algorithm is given belowi.**InputParameters**: Maximum number of iterations max ${Iter}_{max}$, local search parameters such as local ${Iter}_{local,}$ and δ, and the size of the population Nii.**Initialize:** set the iteration counter 1 = t, initialize the number of S_t_ uniformly in X, and identify the best point in S_t_iii.while t < ${Iter}_{max}$ doiv.  $F_{i}^{t}\leftarrow CalcF\left(S_{t}\right)$v.  $y_{i,t}\leftarrow Move(x_{i,t},F_{i}^{t})$vi.  $z_{i,t}\leftarrow Local({Iter}_{local,}\delta ,y_{i,t})$vii.  $x_{i,t+1}\leftarrow Select(S_{t+1},y_{i,t},z_{i,t)}$viii.end while

Step 1: The algorithm runs for ${Iter}_{max}$ iterations or generations; $n\times {Iter}_{local}$ is the maximum number of locations $z_{i,t}$.

Step 2: The points $x_{i,t}$, t = 1 are selected uniformly in X, i.e., $x_{i,t}\;in\;Unif(X)$, i = 1,2,…, N where Unif represents the uniform distribution. The cost function $f(x_{i,t})$ is computed at each iteration and the best point is identified as follows:

(17) $$x_{t}^{B}=\arg max\left\{f(x_{i,t})\right\}\;where\;x_{i,t}\in S_{t}$$

From Equation (17), $x_{t}^{B}$ is the element of $S_{t}$ that gives the maximum numerical value in terms of the fitness function or objective function f.

Step 3: while t < ${Iter}_{max}$ do

Step 4: At this step, a value $\left(q_{i,t}\right)$ is assigned to each point $x_{i,t},$ the charge $q_{i,t}$ of $x_{i,t}$ depends on the function $f\left(x_{i,t}\right)$ and the points which have the best cost function have more charge than other points. At every point, the charges can be computed by Equation (18) as given below:

(18) $$q_{it}=exp\left[-n\frac{f\left(x_{i,t}\right)-f(x_{t}^{B})}{\sum_{j=1}^{N}f\left(x_{i,t}\right)-f(x_{t}^{B})}\right]$$

Then, at this point, the force $F_{i,j}^{t}$, connecting two points $x_{i,t}$ and $x_{j,t},$ can be found by using Equation (19).

(19) $$F_{i,j}^{t}=(x_{j,t}-x_{i,t})\frac{q_{j,t}.q_{i,t}}{{{||x}_{j,t}-x_{i,t}||}^{2}}\;if,\;f(x_{i,t}>x_{j,t})$$

In the end, the total force $F_{i}^{t}$ computed at each $x_{i,t}$ is

(20) $$F_{i}^{t}=\sum_{j=1,j\neq i}^{N}F_{i,j}^{t}$$

Step 5: each point $x_{i,t}$ except for $x_{t}^{B}$ is moved along the total force $F_{i}^{t}$ using:

(21) $$x_{i,t}=x_{i,t}+\lambda \frac{F_{i}^{t}}{{||F}_{i}^{t}||}\left(RNG\right),i=1,2,\ldots ,N,i\neq B$$

where λ in Unif(0,1) for each coordinate of $x_{i,t},$ and RNG is the range of movement toward the upper or lower limits.

Step 6: For each, $y_{i,t}$ a maximum of local ${Iter}_{local,}$ points are generated in each coordinate direction in the δ neighborhood of $y_{i,t}$. This means that the process of generating local points is continued for each $y_{i,t}$ until either a better $z_{i,t}$ is found or the $n\times {Iter}_{local}$ the trail is reached.

Step 7: $x_{i,t+1}\epsilon S_{t+1}$ are chosen from $y_{i,t}$ and $z_{i,t}$ by using Equation (20), and the best solution is recognized by using Equation (21).

The significant steps of the EMO algorithm are given in [8] and the EMO algorithm needs a smaller number of iterations to generate solutions for complex nonlinear optimization problems.

Table 1 depicted the comparative parameters and expressions used for evaluating the proposed method. The main reason for selecting the EMO is, that it gives much better results, as shown in Table 2, Table 3, Table 4, Table 5, Table 6, Table 7, Table 8, Table 9, Table 10, Table 11, Table 12, Table 13, Table 14, Table 15, Table 16 and Table 17. The EMO has been used for solving various optimization problems, including image-processing tasks such as multilevel thresholding. EMO is known for its efficiency in solving complex optimization problems. In the context of multilevel thresholding, EMO can efficiently search for the optimal set of thresholds that maximize the image segmentation quality. EMO is a population-based algorithm that can search the entire solution space and avoid getting stuck in local optima. This is important for multilevel thresholding because the optimal set of thresholds may be located in a complex and highly nonlinear search space. EMO can be easily adapted to handle different types of objective functions and constraints. EMO is robust to noise; in the context of multilevel thresholding, it can handle images with different levels of noise and variability. EMO requires a few parameters to be tuned, which makes it easy to use.

## 3. Energy Curve

To find effective optimized threshold levels, the energy curve [3] will be used instead of the histogram of an image for various applications.

### 3.1. Equation of Energy Curve

Consider an image indicated as I=x(i,j) where i and j are spatial coordinates, i=1,2,…N and j=1,2,...M and the size of the image are X=M×N. For an image, spatial correlation among neighboring pixels can be devised by defining the neighborhood system with N of order d, for an image with spatial coordinates (i,j) as $N_{ij}^{d}=\left\{\left(i+u,j+v\right),(u,v)\in N^{d}\right\}$; various configurations of the neighborhood are described in [30]. Neighborhood systems with second-order are measured for the generation of energy curve, i.e.,$(u,v)\in \left\{\left(\pm 1,0\right),\left(0,\pm 1\right),\left(1,\pm 1\right),(-1,\pm 1)\right\}$.

The foremost step is to find the energy of each pixel value of the entire grayscale range of an image considered; generate a binary matrix $B_{x}=\left\{b_{ij},1\leq i\leq M,1\leq j\leq N\right\}$; $b_{ij}=1$ if $x_{ij}>x$, else $b_{ij}=-1$. Let $C=\left\{c_{ij},1\leq i\leq M,1\leq j\leq N\right\}$ be another matrix, where $c_{ij}=1$,∀(i,j). At each pixel value x, the energy value $E_{x}$ of the image, I can be computed with the below expression.

(22) $$E_{x}=-\sum_{i=1}^{M}\sum_{j=1}^{N}\sum_{pq\in N_{ij}^{2}}b_{ij}b_{pq}+\sum_{i=1}^{M}\sum_{j=1}^{N}\sum_{pq\in N_{ij}^{2}}c_{ij}c_{pq}$$

**Figure 1:**
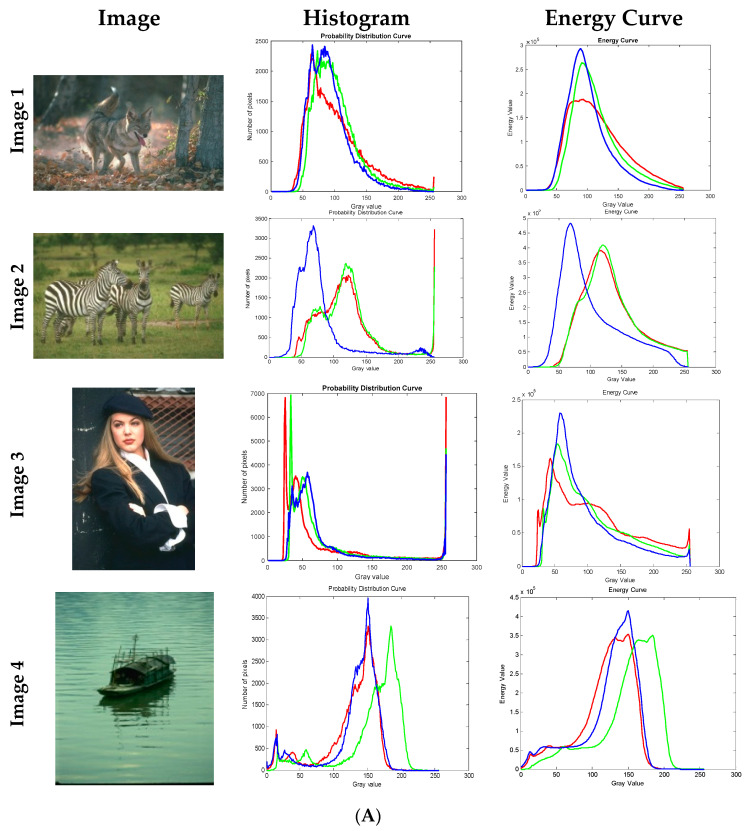

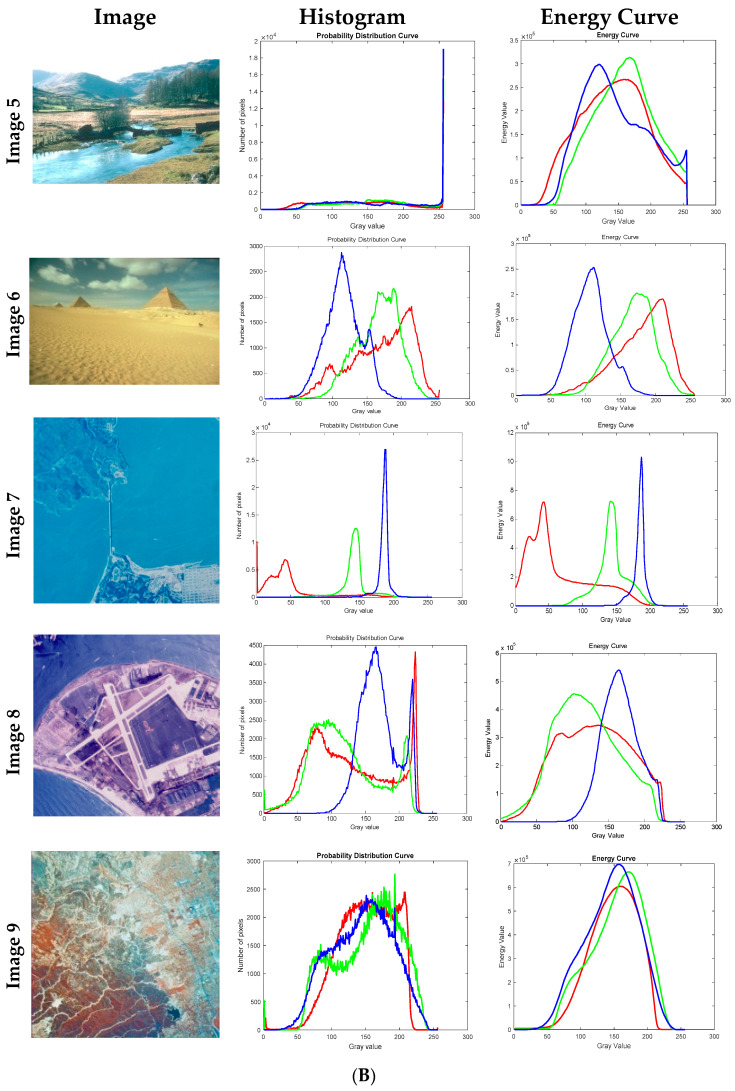
(**A**) Images considered for experimentation along with histogram and energy curves of images. Red, green, and blue color plots indicate histograms and energy curves of red, green, and blue components of input images. (**B**) Images considered for experimentation along with histogram and energy curves of images. Red, green, and blue color plots indicate the histograms and energy curves of red, green, and blue components of input images.

From Equation (22), its second term should be a constant; consequently, the energy associated with each pixel is $E_{x}\geq 0$. From the above equation, we can see that the energy for a particular gray level is zero if each element of $B_{x},$ either 1 or −1 can be put forward in another way as all the pixels of an image I(i,j) with gray level either greater than x or less than x, otherwise, the energy level at a particular gray value x is positive as given in Figure 1.

### 3.2. Characteristics of Energy Plot

The energy plot generated as per Equation (22) is associated with some exciting characteristics. Each object in an image is represented by a gray level range, for instance, the pixel range $\left[t_{1},t_{2}\right]$ represents an object in a given image, at $x=t_{1};$ the elements in $B_{x}$ are 1 for pixels corresponding to the object in the same image. As x increases few elements in the matrix $B_{x}$ will become −1, at $x=t_{2}$; all the matrix elements in $B_{x}$ corresponding to pixels in the object becomes −1. The energy curve produced for the gray-level range $\left[t_{1},t_{2}\right]$ is a bell shape. Figure 1 depicts the image histogram and energy curve related to eight images. The valley and peak points on the energy curve are useful to identify objects in an image.

## 4. Proposed Method

**Figure 2:**
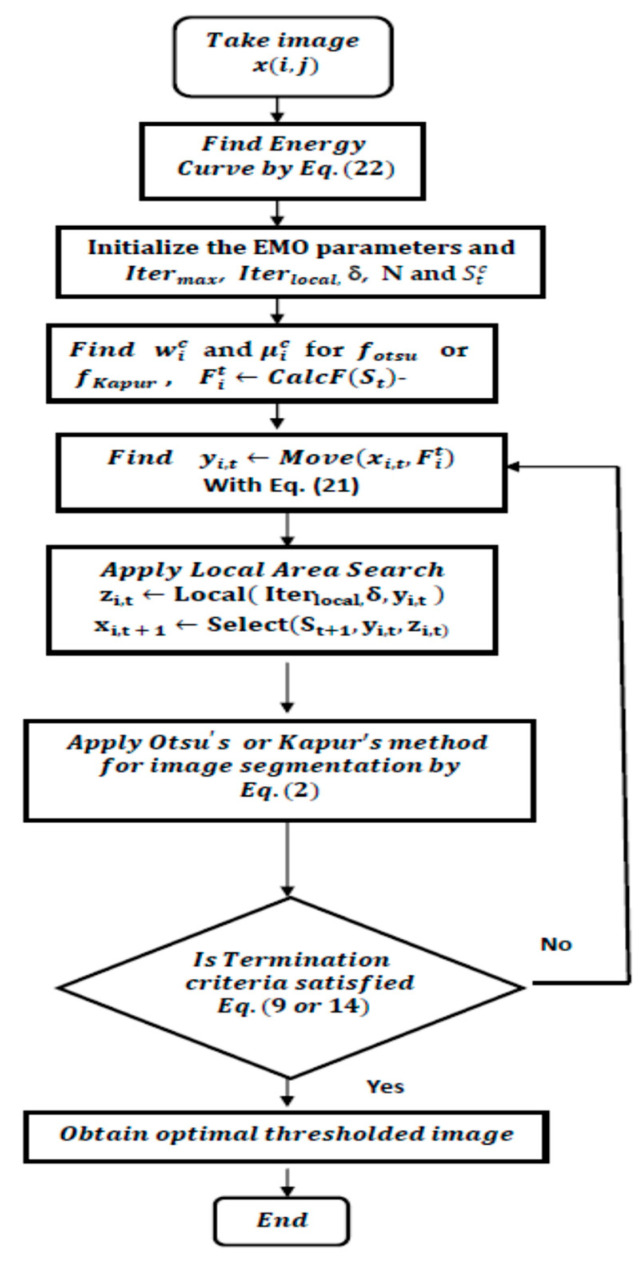
Flow chart of the proposed approach to color image segmentation.

The variety of multilevel thresholding techniques for image segmentation is given in the introduction section and the limitations of the histogram-based techniques are also presented. The proposed method uses an energy curve instead of the histogram, and EMO was used to find optimized threshold levels on the energy curve by maximizing the inter-class variance and entropy for Otsu’s method and Kapur’s method, respectively, as given in Equation (11) for Otsu’s method; the flow chart of a new approach is given in Figure 2.

From the flow chart, take an image for experimentation x(i,j) for multilevel thresholding-based segmentation and plot the energy curve of the considered color image by using Equation (1), then assign the design parameters of EMO and the solution matrix values are filled with arbitrary numbers, initially denoted as $x_{i}$ (set of threshold levels) as per Equation (18), then divide all the pixels in the image as per selected threshold levels into different classes or regions as per Otsu’s technique and Kapur’s method, then find the inter-class variance and entropy of the segmented image, as given in Equation (11). Afterward, find the new set of threshold levels with Equation (17) again, find the fitness and compare it with the previous fitness function, and run this procedure until there is no improvement in the objective function or the specified number of iterations is reached, and lastly find the optimized threshold valued ($x_{new}$) and classify the gray levels as Equation (3) for final segmentation for R, G, and B components separately for color images. The results of this method are compared with histogram-based techniques for evolution.

Steps in the implementation of the proposed method for color image segmentation are given in Table 18 below.

The Algorithm for EMO initialization is given below as Algorithm 2.
**Algorithm 2:** EMO initialization1.**For** i = 1 to m **do**2.   **for** k = 1 to d **do**3.           
λ←rand(0,1)
4.           
$x_{k}^{i}\leftarrow l_{k}+\lambda (u_{k}-l_{k})$
5.   **end for**6.**Compute** $f(X^{i})$7.**End for**

The Algorithm to find optimized or best threshold values is given below as Algorithm 3.
**Algorithm 3:** Find optimized threshold values1.count←12.$Length\leftarrow \delta (max(u_{k}-l_{k}))$3.**For** i = 1 to m **do**4.**for** k = 1 to d **do**5.$\lambda_{1}\leftarrow rand(0,1)$6.**while** count < LSITER **do**7.$y\leftarrow x^{i}$8.$\lambda_{2}\leftarrow rand(0,1)$9.    **if** $\lambda_{1}>0.5$ **then**10.    $y_{d}=y_{d}+\lambda_{2}\cdot (Length)$11.    **else**12.    $y_{d}=y_{d}-\lambda_{2}\cdot (Length)$13.    **end if**14.    $iff\left(y\right)<f(X^{i})$ **then**15.      $x^{p}\leftarrow y$16.      count←LSITER−117.    **end if**18.    count←count+119.    **End while**20.    **end for**21.**end for**22.$X^{best}\leftarrow \arg\min(f(x^{i})),x^{i}\in X$

From the above algorithms, pseudo-code, LSITER is the number of local search iterations. The steps given in Algorithms 2 and 3 can be treated as pseudo-code also.

**Figure 3:**
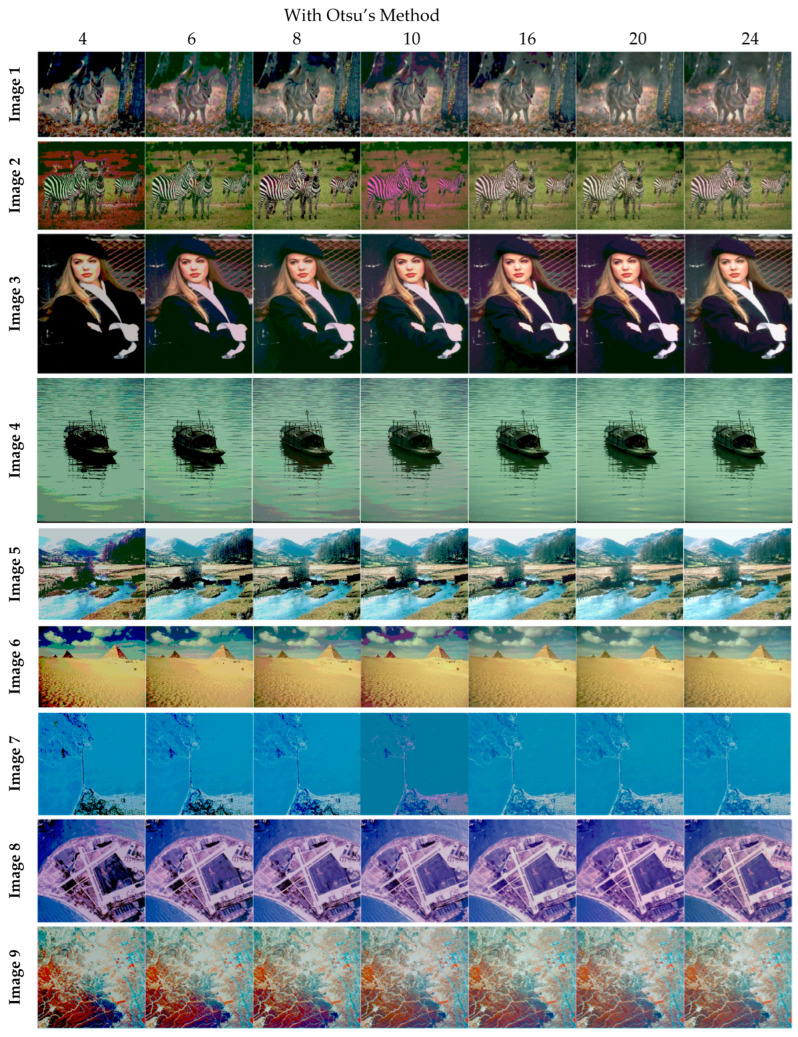
Segmented images of Image 1 to Image 9 for N = 4, 6, 8, 10, 16, 20, and 24 using the proposed method based on Otsu’s method.

**Figure 4:**
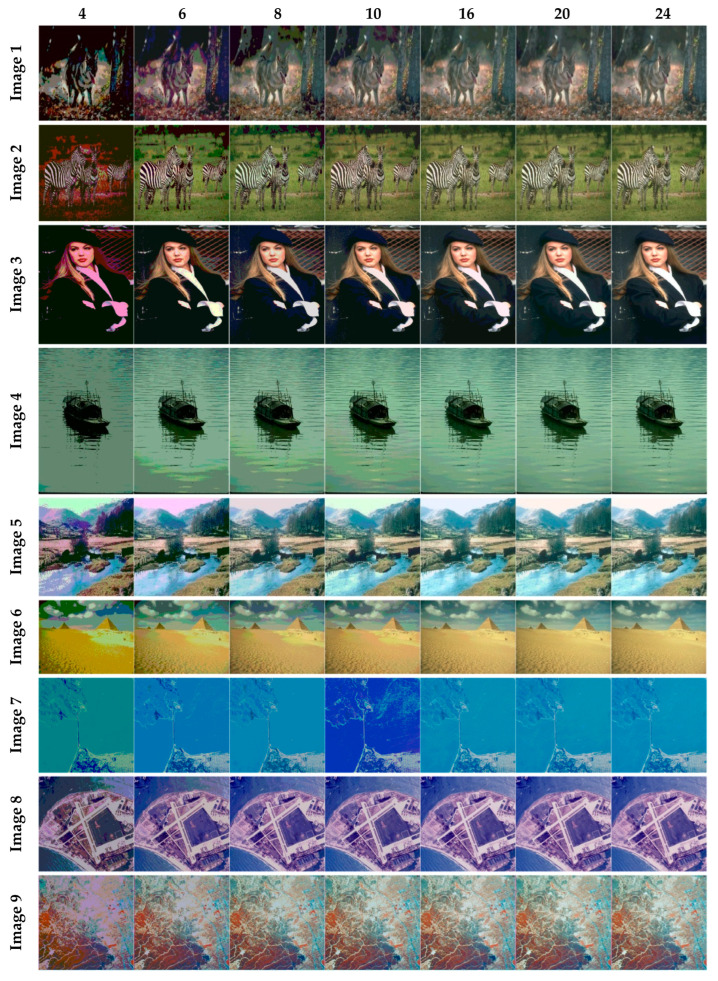
Segmented images of Image 1 to Image 9 for N = 4, 6, 8, 10, 16, 20, and 24 using the proposed method based on Kapur’s method.

**Figure 5:**
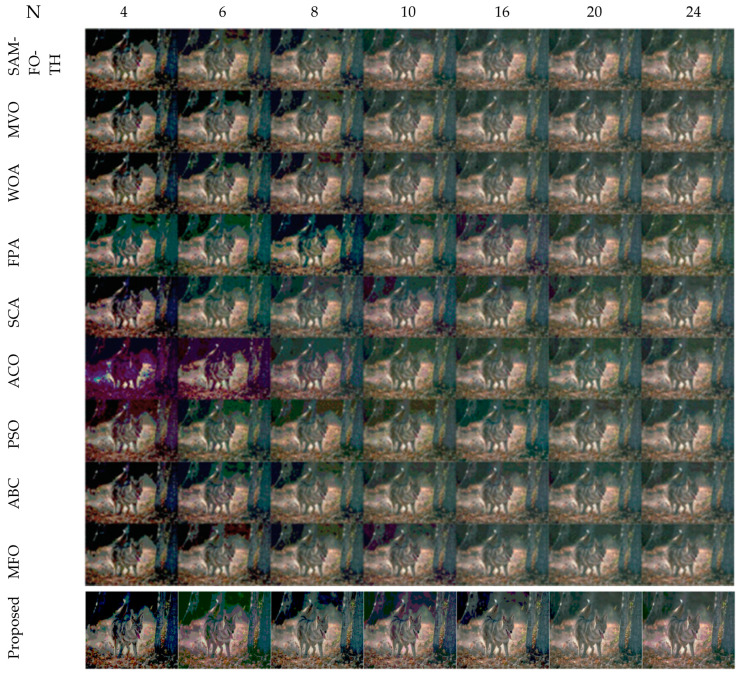
Segmented images of Image 1 at N = 4, 6, 8, 10, 16, 20, and 24, using SAMFO-TH, MVO, WOA, FPA, SCA, ACO, PSO, ABC, and MFO, and with the proposed model based on Kapur’s method.

**Figure 6:**
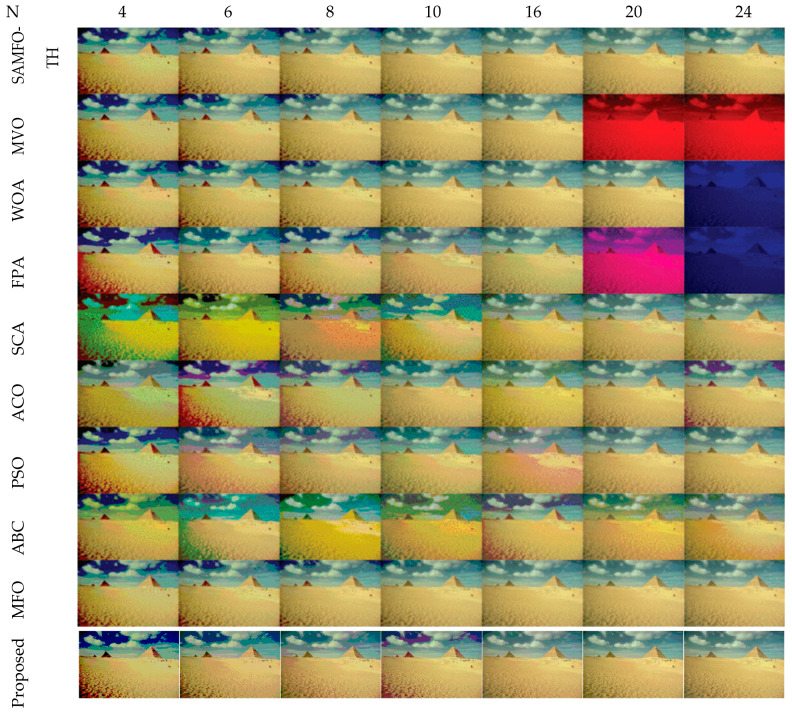
Segmented images of Image 6 at N = 4, 6, 8, 10, 16, 20, and 24, using SAMFO-TH, MVO, WOA, FPA, SCA, ACO, PSO, ABC, and MFO, and with the proposed model based on Otsu’s method.

**Figure 7:**
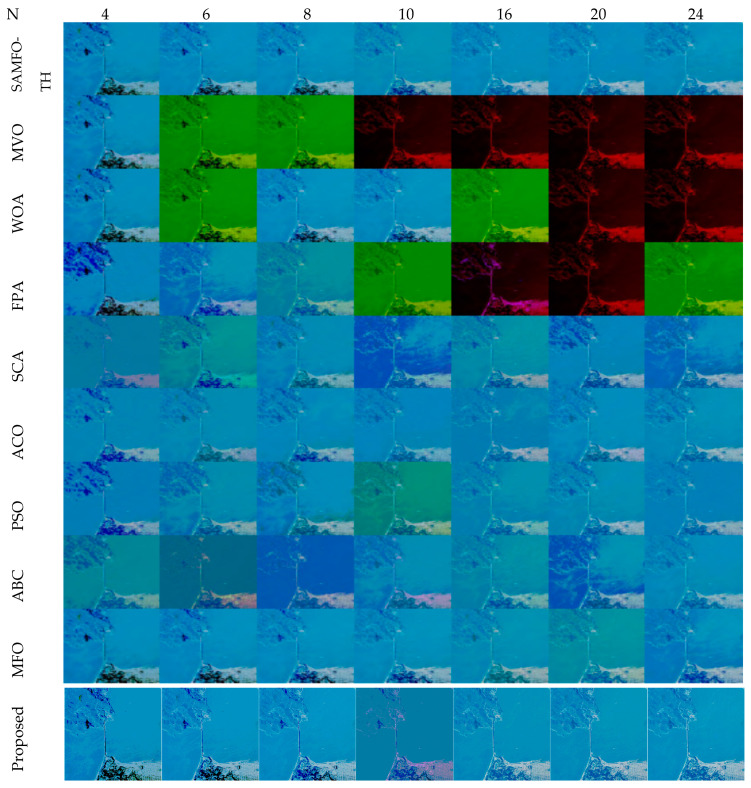
Segmented images of Image 7 at N = 4, 6, 8, 10, 16, 20, and 24, using SAMFO-TH, MVO, WOA, FPA, SCA, ACO, PSO, ABC, and MFO, and with the proposed model based on Otsu’s method.

**Figure 8:**
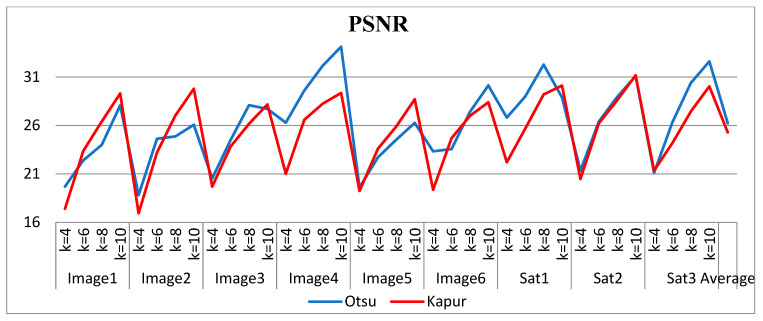
Comparison of PSNR for the proposed method using Otsu’s and Kapur’s methods.

**Figure 9:**
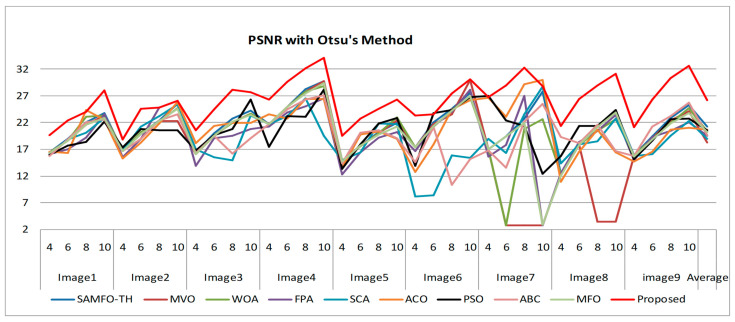
Comparison of PSNR based on Otsu’s method.

**Figure 10:**
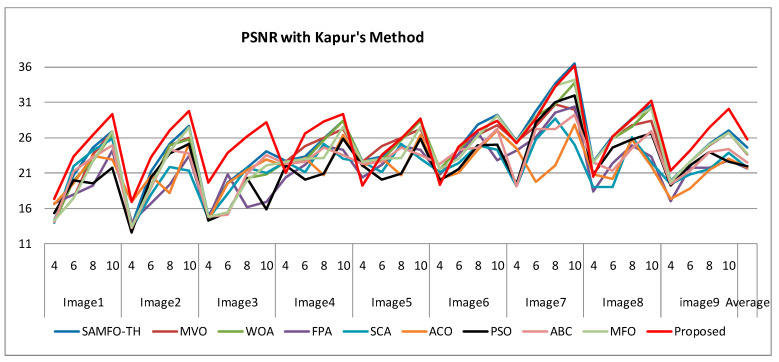
Comparison of PSNR based on Kapur’s method.

**Figure 11:**
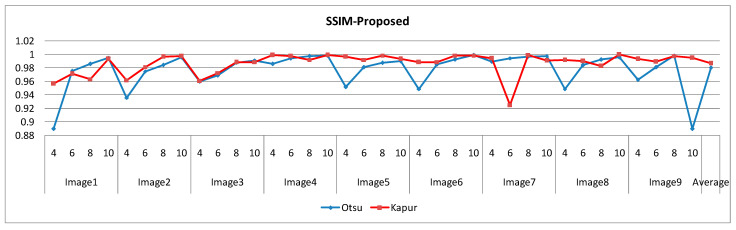
Comparison of SSIM with the proposed method based on Otsu’s and Kapur’s criteria.

**Figure 12:**
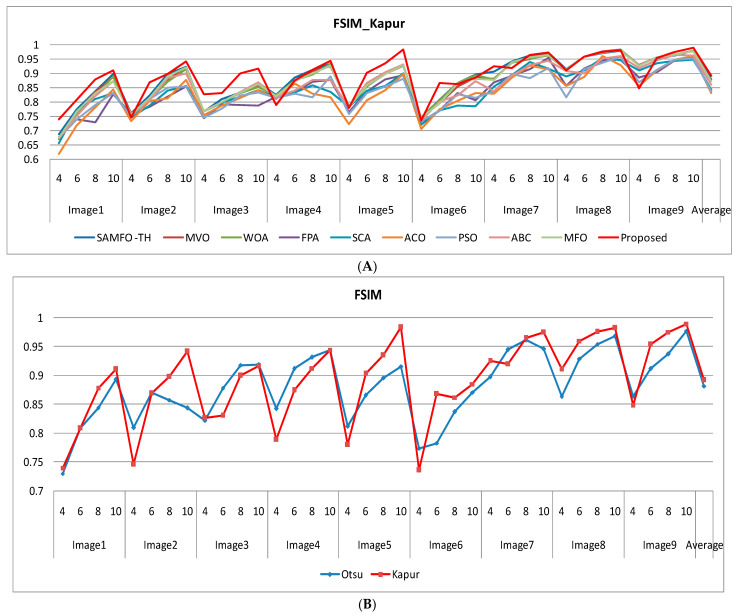
(**A**) Comparison of PSNR based on Kapur’s method. (**B**) Comparison of FSIM with the proposed method based on Otsu’s and Kapur’scriteria.

The proposed “multilevel thresholding based on EMO and energy curve (MTEMOE)” has many advantages over other methods for natural color images as illustrated in Table 2, Table 3, Table 4, Table 5, Table 6, Table 7, Table 8, Table 9, Table 10, Table 11, Table 12, Table 13, Table 14, Table 15, Table 16 and Table 17 and Figure 3, Figure 4, Figure 5, Figure 6, Figure 7, Figure 8, Figure 9, Figure 10, Figure 11 and Figure 12. Despite its merits, the MTEMOE method also has some limitations such as being based on an energy curve, which takes more time compared to the time needed to compute the histogram of an image. Direct keywords for computing the histogram of an image are available in Matlab and other scientific languages but the code required to generate the energy curve needs to be developed by researchers based on Equation (22). In the case of color image segmentation, the time taken to compute is much greater than the energy curve that needs to be computed for three color components of the image. While EMO has been successfully applied to a wide range of optimization problems, it also has some limitations. Multilevel thresholding of images often involves optimizing over high-dimensional search spaces, which can make it difficult for EMO to converge to an optimal solution in a reasonable amount of time. Images may contain noise that can affect the performance of EMO. EMO may not be able to handle the noise and may converge to suboptimal solutions. EMO may not be adaptable to different types of images, such as images with varying contrast or illumination.

The advantage of context-sensitive multilevel thresholding with an energy curve can be used with different upcoming new optimization techniques to further improve the effectiveness of segmentation. This method proposed with electromagnetic optimization can be extended for color images with different sorts of artifacts and can be tested for its efficiency. EMO with an Energy Curve can be applied to other image-processing tasks, such as image denoising, image compression, and image restoration. Hybrid optimization algorithms can be developed that combine EMO with other optimization techniques to further improve the performance of multilevel thresholding. The robustness of EMO can be studied for multilevel thresholding by testing it on a variety of images with different characteristics, such as size, complexity, and noise levels. Future research work in this area has the potential to contribute to the development of more efficient and effective algorithms for image-processing tasks.

## 5. Results and Discussions

This section describes the experimental results of the proposed method and compares it with existing state-of-the-art techniques, and also explains the source of images under test and metrics used for the evolution of the segmentation techniques.

The proposed algorithm and existing techniques are experienced with color images fetched from USC-SIPI and Berkeley segmentation data set (BSDS500); a total of nine images are considered for the test, six natural images and three satellite images as shown in Figure 1; in the same image the histograms and energy curves are also illustrated, indicated as Images 1–9; all of the images considered for experimentation have distinct features. In this study mainly objective analysis is adapted and depends on numerical values instead of quality measures based on visual perception [40].

The comparative analysis between the proposed algorithm and other different optimization algorithms such as SAMFO -TH [9], MVO [29], WOA [24], FPA [28], SCA [25], ACO [16], PSO [13], ABC [19], and MFO [21] is necessary. The results and experimental setups are taken from published articles [8] to compare with the proposed method, all the algorithms executed until there is no change in the fitness function, and the MEAN value fitness function of all the algorithms [8] is illustrated in Table 17. All the images are tested with the number of threshold levels N = 4, 6, 8, 10, 16, 20, and 24.

The selection of comparative metrics [8] is an important task; it should be done in such a way as to test all the aspects of segmentation. The parameters used in this study [4] are described in this section. (i) The mean value of fitness (MEAN) with Kapur’s and Otsu’s method, is considered a significant metric to test the performance of optimization schemes. This index is computed using Equation (9) in Otsu’s method or Equation (3) in Kapur’s entropy. It demonstrates the robustness of the optimization algorithm in the course of selecting the optimized threshold vector. (ii) Peak signal-to-noise ratio (PSNR), this parameter estimates the deviation of a segmented image from its original image, which indicates the quality of a reconstructed image. A high PSNR value refers to better segmentation. (iii) Mean square error (MSE), a lower MSE value illustrates better segmentation; it computes the average of the square of the error. (iv) Structural similarity (SSIM), this parameter gives the level of similarity between the segmented and input image under test; a greater value of SSIM [39] indicates a better segmentation effect; it is in the range from −1 to +1. (v) Feature similarity (FSIM), this is similar to SSIM, which indicates degradation of image quality; it ranges [−1, 1]; a high value of FSIM means better segmentation of the color image. (vi) probability Rand index (PRI) or simply Rand index (RI), this computes the connection between the ground truth and segmented image; better performance [9,42,43] is indicated by a higher PRI value. (vii) Variation of information (VOI), this gives the randomness of a segmented image; a low VOI value indicates better segmentation performance. All comparative parameters are described along with the required equations in Table 1. The segmented images with various optimization techniques are obtained from published articles and this study proves that the proposed approach provides better performance [44,45] than the techniques considered in this research work. Figure 2 and Figure 3 illustrate the segmented results using the proposed (MTEMOE) approach to color image segmentation based on Otsu’s and Kapur’s methods [43,46,47]. In the end, a statistical analysis is firmly used to demonstrate the dominance of the proposed approach. The segmented images are depicted in Figure 2, Figure 3, Figure 4, Figure 5, Figure 6 and Figure 7 for threshold levels = 4, 6, 8, 10, 16, 20, and 24 using Otsu’s variance and Kapur’s entropy. Figure 2 illustrates the segmented resultant images with the proposed image with Kapur’s methods; at the same time, segmented results of the proposed technique are given in Figure 3, Figure 4 and Figure 5 with a focus on results with SAMFO-TH, MVO, WOA, ABC, MFO, ACO, and ABC based on Kapur’s entropy as the fitness function. Figure 6 and Figure 7 demonstrate results with Otsu’s methods with the above-mentioned optimization techniques. The comparative metrics of segmentation performance are presented in Table 2, Table 3, Table 4, Table 5, Table 6, Table 7, Table 8, Table 9, Table 10, Table 11, Table 12, Table 13, Table 14, Table 15, Table 16 and Table 17; the performance parameters used are MEAN, PSNR, MSE, SSIM, FSIM, PRI, andVoI.

The required expressions of comparative parameters are given in Table 1. From Table 2, Table 3, Table 4, Table 5, Table 6, Table 7, Table 8, Table 9, Table 10, Table 11, Table 12, Table 13, Table 14, Table 15, Table 16 and Table 17, the values of comparative metrics are presented for the proposed method and another existing method. In Table 17, the average MEAN values of fitness with Kapur’s and Otsu’s methods on optimization techniques MVO, WOA, PFA, SCA, ACO, PSO, ABC, MFO, and SAMFO-TH, and for the proposed approach on nine images considered with threshold levels N = 4, 6, 8, and 10 are given. It shows clearly that the proposed methods result in higher values of average MEAN with both Kapur’s and Otsu’s techniques. The average MEAN values of fitness are computed separately for three color components (R, G, and B) for each image. In particular, the values with the proposed method with Otsu’s techniques are much higher compared with other optimization techniques. In Table 2 PSNR values are presented for SAMFO-TH, MVO, WOA, FPA, SCA, ACO, PSO, ABC, and MFO, and the proposed model using Kapur’s method with threshold levels N = 4, 6, 8, and 10; the results clearly show that the PSNR values with the proposed method are much better than other techniques, especially with N= 10. In Table 9, PSNR values with Otsu’s method are given and with all the images the PSNR values for the proposed method are superior to any other method considered; the average PSNR with the proposed method is 26.2278 which is higher than other techniques; after the proposed method, the SAMFO-TH method gives the best PSNR values. In Table 3 and Table 10, the mean square error (MSE) values with Kapur’s and Otsu’s techniques are given for the proposed method and other techniques. The required expression to compute MSE is mentioned in Table 1. MSE value should be less for better segmentation; the MSE values are much less for the proposed method compared to other techniques, especially for higher thresholding levels (8 and 10). From Table 10, the average MSE for all nine images is 229.5213 with the proposed methods, whereas its value is 1449.4559 with SCA-based segmentation. After the proposed method, the SAMFO-TH provides the best MSE values with both Kapur’s and Otsu’s techniques. In Table 4 and Table 11, the structural similarity index (SSIM) is given for Kapur’s and Otsu’s techniques; its value should be higher for better segmentation. The value of SSIM with the proposed method is slightly higher than the SAMFO-TH method but much higher than multilevel thresholding techniques with other optimization methods considered for comparison.

In Table 5 and Table 12, the featured similarity index (SSIM) is given for Kapur’s and Otsu’s techniques; its value should be higher for better segmentation. The value of FSIM with the proposed technique is higher than all other techniques. The average FSIM computed for nine images with the proposed technique with Otsu’s method is 0.8818; its value with SCA is only 0.8011. From Table 6 and Table 13, the PRI should be a higher value for better image segmentation. The PRI values are slightly better for SAMFO-TH compared to the proposed method, whereas its values are much better than other techniques.

In Table 15 and Table 16, there is a comparison of MEAN computed by SAMFO-TH, MVO, and WOA using Otsu’s and Kapur’s methods with N = 4, 6, 8, and 10 for red, green, and blue components separately; its values are much higher with Otsu’s method than Kapur’s method. After analyzing the information from Table 17 it can be concluded that the proposed method gives a much better average MEAN of fitness with both Kapur’s and Otsu’s methods than all other techniques considered.

In this discussion of results, the proposed approach is compared with other algorithms using the mean of fitness function (MEAN); in Table 8, the MEAN values computed by the proposed method are given for both Kapur’s and Otsu’s methods. Higher MEAN values indicate higher accuracy. These values are significantly higher than those values obtained with other methods, including SAMFO-TH; as the level of threshold increases the MEAN values increase in both Kapur and Otsu methods. The MEAN values are much greater with Otsu’s method than with Kapur’s method. Table 15 depicts the MEAN values with SAMFO_TH, MVO, and WOA with Otsu’s methods; these values are much lower than with the proposed approach and MEAN values with other optimization techniques can be fetched from published [8] articles for comparison. In Table 16, a comparison of MEAN computed by SAMFO-TH, MVO, and WOA using Kapur’s method with N = 4, 6, 8, and 10 for the red, green, and blue components is given. Very importantly, in Table 17, the average of MEAN values with various optimization techniques with both Otsu’s and Kapur’s methods are presented; the results show that the results with the proposed method are highly superior to all the techniques considered in this research, for color components red, green, and blue. From Table 17, we can conclude that the mean of MEAN value for all the images is higher with the proposed approach with both Otsu’s and Kapur’s methods; at the same time the performance of SCA, PSO, and MFO is not up to the mark; after the proposed approach SAMFO-TH is the best one. From this discussion, we can conclude that the proposed approach for segmentation performs with better stability.

In Table 1 PSNR values with Kapur’s method are presented for all the optimization techniques which are under test and, in Table 9, PSNR values with Otsu’s method are given. From the two tables mentioned above, we can deduce the conclusion that the proposed approach produces better PSNR compared to other methods; PSNR performance is much higher with Kapur’s than with Otsu’s method, as the level thresholding increases PSNR also increases tremendously. The mean PSNR for nine images with the proposed method is 25.2768 (from Table 2) and 24.6188 with SAMFO-TH; the lowest value is with FPA at 21.678. At the same time, the mean of PSNR with Otsu’s criteria is 26.222 for the proposed method, 21.2768 with SAMFO-TH, and the lowest value is 19.5712 with FPA. PSNR values are higher for satellite images (Images 7, 8) compared to the rest of the images; for Image 3 PSNR performance is very low; from the above discussion, the proposed method can provide better PSNR compared to the other methods considered. Lower MSE implies better segmentation performance; from Table 3 and Table 10, MSE with the proposed approach is much lower than with other methods for Kapur’s and Otsu’s techniques. The average MSE value with the proposed method is 294.4714, whereas it is 707.477 with FPA for Kapur’s method.

Other most significant quality metrics for color image segmentation are SSIM and FSIM, and higher values of FSIM and SSIM indicate accurate image segmentation. In Table 4, SSIM values are presented and computed by SAMFO-TH, MVO, WOA, FPA, SCA, ACO, PSO, ABC, and MFO, and with the proposed model using Kapur’s method with N = 4, 6, 8, and 10. In Table 11, a comparison of SSIM with Otsu’s method is described; mean values of SSIM for the technique are given which indicate the overall SSIM performance of nine images. For instance, from Table 4, SSIM is 0.9867 with the proposed method and 0.98539 with SAMFO-TH, with only slight variation with other methods. In Table 5 and Table 12, FSIMs with Kapur’s and Otsu’s methods are presented, respectively. From Table 5, they are 0.8923 for the proposed method, 0.7898 with SAMFO-TH, and finally, the lowest value is 0.8377 with PSO. Both the SSIM and FSIM values are enhanced along with threshold levels from 4 to 10.

The VOI and PRI are important and distinguishing comparative metrics in the field of segmentation. High-quality segmentation is referred to by higher PRI and low value of VOI. The PRI values with various techniques including the proposed one (MTEMOE) are illustrated in Table 6 and Table 13 with Kapur’s and Otsu’s methods, respectively. From Table 6, the PRI value with the proposed method is better than WOA, FPA, SCA, and ACO, but lower than other methods with Kapur’s method. With Otsu’s criteria, MTEMOE performs well in terms of PRI compared to all the techniques other than SAMFO-TH and WOA, as illustrated in Table 13; finally, we point out that higher PRI values are generated with Otsu’s method compared to Kapur’s method. However, with higher threshold levels (N = 16, 20, and 24) the proposed method gives higher PRI values compared to all other methods considered in this study. From Table 7 and Table 14, the VOIfor the proposed method gives better results than other techniques for both Kapur’s and Otsu’s methods; only WOA and SAMFO-TH give a minute improvement in the case of Otsu’s methods; at a higher level of thresholding, the proposed method gives much lower(or better) values compared with the methods in this study including SAMFO-TH. The overall impression is that the MTEMOE is a better approach to color image segmentation than other state-of-the-art techniques and the proposed technique uses an energy curve instead of a histogram.

## 6. Conclusions

In this article, many schemes for color image segmentation are discussed. From that pool of methods, multilevel thresholding (MT) is a powerful technique, generally based on the histogram of an image. To nullify the shortfalls of the histogram, another curve that is similar to the histogram called the energy curve is used instead of the histogram to efficiently compute optimized thresholds. The proposed model for segmentation is based on Otsu’s and Kapur’s methods for MT on an energy curve with EMO for finding optimized threshold levels by maximizing the inter-class variances and entropy. The results for a group of color benchmark images clearly show that MT on the energy curve is more efficient than the histogram-based techniques. The energy curve can consider spatial contextual information to find energy levels at each pixel. Consequently, the same veiled information is used to compute optimized levels. The efficiency of the proposed approach is evaluated with mean of fitness (MEAN), PSNR, MSE, PRI, VOI, SSIM, and FSIM. The proposed approach (MTEMOE) is tested on nine color images using both Otsu’s and Kapur’s methods at different threshold levels (N = 4, 6, 8, 10, 16, 20, and 24); the proposed method is compared with other state-of-the-art methods for color image segmentation: SAMFO-TH, MVO, WOA, FPA, SCA, ACO, PSO, ABC, and MFO. From the results, we can conclude that the value of PSNR is greater with the energy curve than with methods based on the histogram; for the proposed method, the MEAN of the objective function is very high compared with a histogram-based method with optimization techniques. The higher PRI and lower VOI values mean better inter-class variance with the proposed method. Based on the values of comparative metrics such as PSNR, MSE, VOI, PRI, and the average MEAN value of fitness function and other parameters, the methods for segmentation of a color image are arranged from best to worst as the proposed method, SAMFO-TH, ACO, SCA, PSO, WOA, MFO, ABC, FPA, SCA, and MVO. Finally, we can conclude that the proposed approach gives an overall better performance for color image segmentation than the methods considered for various applications. The energy curve can be used with the latest upcoming optimization algorithms for still better results.

## Figures and Tables

**Table 1:** Different metrics to test the efficiency of the algorithms.

S.No	Comparative Parameters	Formula	Remarks
1	The mean value of fitness (MEAN)	It can be calculated as the average value of fitness values of objective values at each iteration of the algorithm.	Inter-class variance and entropy are the objective functions for Otsu’s and Kapur’s methods.
3	Peak signal-to-noise ratio (PSNR)	$20{log}_{10}{\left(\frac{MAX}{RMSE}\right)}^{2}$	The MAX is the maximum gray value taken as 255.
4	Mean square error (MSE)	$MSE={\left(RMSE\right)}^{2}$ $RMSE=\sqrt{\frac{\sum_{i=1}^{R_{0}}\sum_{j=1}^{C_{0}}{\left(I\left(i,j\right)-I^{s}\left(i,j\right)\right)}^{2}}{R_{0}\times C_{0}}}$	I(i,j) is the input image and the segmented image is $I^{s}\left(i,j\right)$. $C_{0}\times R_{0}$ is the size of the image.
5	Structural similarity (SSIM)	$=\frac{\left(2\mu_{x}\mu_{y}{+c}_{1}\right)(2\sigma_{xy}+c_{2})}{\left(\mu_{x}^{2}+\mu_{y}^{2}{+c}_{1}\right)(\sigma_{x}^{2}+\sigma_{y}^{2}{+c}_{2})}$	$\mu_{x}$ and $\mu_{y}$ are the mean intensities of input and segmented images. $\sigma_{xy}$ is the covariance, $\sigma_{x}^{2}$ and $\sigma_{y}^{2}$ are the variance of images.
6	Feature similarity index (FSIM)	$=\frac{\sum_{x\epsilon \Omega }S_{L}\left(x\right){PC}_{m}(x)}{\sum_{x\epsilon \Omega }{PC}_{m}(x)}$	$S_{L}\left(x\right)$ is the similarity between images. ${PC}_{m}(x)$ is the maximum phase congruency of two images.
7	Probability Rand Index (PRI)	The internal validation measurePRIis an indication of resemblance between two regions in an image or clusters; it is expressed as given below $PRI=\frac{a+b}{a+b+c+d}$	Whereas dataset X is portioned into two subsets $C_{1}$ and $C_{2}$, the number of pairs of pixels (or elements) that are present in both subsets $C_{1}$ and $C_{2}$ is indicated by a. b indicates the number of pairs of elements in X that are a different subset in C1 and a different subset in C2. c indicates the number of pairs of elements in X that are the same subset in C1 and a different subset in C2. d denotes the number of pairs of elements in X that are a different subset in C1 and the same subset in C2.
8	Variation of information (VOI)	$VOI=Ent\left(I^{s}\right)+Ent\left(I^{R}\right)-2MI(I^{s},I^{R})$ $MI\left(I^{s},I^{R}\right)=Ent\left(I^{s}\right)+Ent\left(I^{R}\right)-Ent(I^{s},I^{R})$	$I^{s}$ is the segmented image, $I^{R}$ is the reference image, Ent is entropy, $Ent(I^{s},I^{R})$ is joint entropy.

**Table 2:** Comparison of PSNR computed by SAMFO-TH, MVO, WOA, FPA, SCA, ACO, PSO, ABC, and MFO with the proposed model using Kapur’s method with N = 4, 6, 8, and 10.

Image	N	PSNR									
		SAMFO-TH	MVO	WOA	FPA	SCA	ACO	PSO	ABC	MFO	Proposed
Image 1	4	14.2867	14.2850	14.1218	16.6767	13.9373	16.6382	15.3040	14.1319	14.2850	17.411
	6	0.7623	17.4474	20.0144	18.0251	22.0077	19.5418	19.9410	21.2326	17.4474	23.3624
	8	24.5806	23.6349	22.7130	19.2523	24.1795	23.4068	19.5648	23.2057	22.6762	26.4105
	10	26.9502	26.7858	26.4701	24.2091	25.8698	22.9264	21.8110	24.9282	26.8866	29.3038
Image 2	4	13.5789	13.3799	13.2111	14.0750	13.1322	16.9569	12.6058	13.2116	13.3799	16.9332
	6	21.2486	19.1958	19.1717	16.7705	18.0188	20.6228	20.3663	18.9082	19.2011	23.2135
	8	25.1726	24.9161	24.9029	19.3409	21.8487	18.1980	23.7460	24.3227	24.1126	27.0255
	10	27.6556	26.0105	25.7441	23.4190	21.3457	25.1496	25.1499	23.7446	27.4677	29.7932
Image 3	4	14.9730	14.7516	14.7516	14.6601	14.6416	14.3734	14.3436	14.7839	14.8017	19.6832
	6	19.6315	15.3583	15.3563	20.8057	18.0842	19.5661	15.4180	15.1249	15.4616	23.8255
	8	21.8110	20.2832	20.2763	16.1667	21.7836	21.4141	20.3278	20.8963	19.9967	26.1728
	10	24.0922	20.8407	20.7880	16.9484	21.0673	22.9669	15.8518	23.5685	22.0379	28.1644
Image 4	4	22.6650	22.6187	22.6187	20.3850	22.5610	21.9843	22.1260	22.2906	22.5522	21.0083
	6	23.3561	24.8435	22.8323	22.1698	21.1181	23.1694	20.0681	22.5925	23.0749	26.5867
	8	25.8717	25.9515	25.6615	24.8007	25.1022	20.6755	20.9384	24.5891	23.1128	28.2479
	10	28.3709	27.2012	28.3390	24.2414	22.9892	26.3917	25.8571	23.5946	27.5801	29.3581
Image 5	4	22.6650	22.6187	22.6187	20.3850	22.5610	21.9843	22.1260	22.2906	22.5522	19.2267
	6	23.3561	24.8435	22.8323	22.1698	21.1181	23.1694	20.0681	22.5925	23.0749	23.5926
	8	25.8717	25.9515	25.6615	24.8007	25.1022	20.6755	20.9384	24.5891	23.1128	25.8969
	10	28.3709	27.2012	28.3390	24.2414	22.9892	26.3917	25.8571	23.5946	27.5801	28.7066
Image 6	4	21.5290	21.5290	21.5290	20.8569	21.0864	20.1992	19.9682	22.2765	21.5068	19.3631
	6	24.3622	24.3605	24.3905	23.6272	22.4015	21.0277	21.5290	24.3142	23.0741	24.6747
	8	27.8633	26.4006	26.2061	26.7374	24.9330	24.2707	24.8640	24.5837	26.2940	26.9835
	10	29.1790	27.7527	29.1194	22.7830	24.2800	27.0600	25.0170	27.2259	29.0730	28.3946
Image 7	4	25.5470	25.4570	25.4570	24.1766	19.1235	24.5240	19.4536	19.0881	25.4570	25.195
	6	29.7362	27.6434	28.9754	26.1089	25.7468	19.7999	28.1928	27.1875	28.9557	28.6755
	8	33.6300	30.7366	30.6802	29.5335	28.6656	22.0921	30.9938	27.1974	33.3217	33.1943
	10	36.4519	29.9769	33.7857	30.3995	24.9723	27.8741	31.9742	29.1914	34.1678	36.1217
Image 8	4	22.6392	22.5244	22.5244	18.4377	19.0066	20.8646	20.8291	22.5279	22.5244	20.4749
	6	26.1655	25.8536	25.8350	22.4239	19.0136	20.1567	24.5617	21.3842	25.8134	26.2102
	8	28.6575	27.7273	27.5732	24.9561	26.0938	25.7541	25.7131	24.5071	28.6420	28.6113
	10	30.5915	28.401	30.2800	23.3037	22.4820	21.9794	26.6112	26.8636	30.2009	31.1743
Image 9	4	19.8193	19.7421	19.7421	17.0760	19.4905	17.2988	19.2776	19.4984	19.8087	21.3238
	6	22.6291	22.5596	22.6157	21.7415	20.7706	18.7977	22.0371	21.3628	22.6060	24.1806
	8	25.1702	24.9523	25.0680	21.7143	21.5330	21.4059	23.9504	23.9585	25.0186	27.439
	10	27.0309	26.7189	26.6886	22.8024	23.8913	23.0109	22.6262	24.3817	26.6501	30.0399
Average		24.6188	23.6325	23.8026	21.6728	21.7482	21.7316	21.9444	22.4984	23.7086	25.7213

**Table 3:** Comparison of MSE computed by SAMFO-TH, MVO, WOA, FPA, SCA, ACO, PSO, ABC, and MFO with the proposed model using Kapur’s method with N = 4, 6, 8, and 10.

Image	N	MSE-Kapur									
		SAMFO-TH	MVO	WOA	FPA	SCA	ACO	PSO	ABC	MFO	Proposed
Image 1	4	2423.32	2424.22	2517.14	1397.72	2626.34	1410.17	1917.33	2511.23	2424.28	1183.26
	6	545.564	1170.47	648.102	1024.60	409.55	722.602	659.140	489.575	1170.47	299.806
	8	197.301	281.571	348.158	772.419	226.473	296.753	718.782	310.824	351.125	148.604
	10	131.237	136.302	146.579	246.702	168.305	331.467	428.533	209.054	133.175	76.3309
Image 2	4	2956.02	2986.04	3104.45	2544.33	3161.27	1310.45	3568.64	3104.05	2986.03	1317.53
	6	355.465	782.530	786.885	1367.80	1026.15	487.773	597.653	836.101	781.568	310.2633
	8	197.617	209.639	210.277	756.819	424.825	984.645	274.458	240.330	252.243	128.9824
	10	111.562	162.940	173.249	295.925	476.991	198.665	198.650	274.547	116.495	68.19623
Image 3	4	2152.35	2177.33	2177.33	2223.71	2233.28	2375.45	1899.97	2151.26	2152.30	699.4559
	6	707.825	1893.42	1894.34	540.146	1010.88	718.566	1867.60	1998.01	1848.93	269.4824
	8	428.524	609.199	610.169	1571.85	431.236	469.541	602.970	528.995	650.742	156.9641
	10	253.433	535.808	542.347	1312.95	508.565	328.393	1690.17	285.908	406.716	99.2294
Image 4	4	352.032	355.804	355.804	595.089	336.500	411.762	316.575	383.729	361.295	515.5257
	6	300.242	213.170	338.727	394.552	502.651	313.429	640.131	357.955	320.322	142.6955
	8	168.231	165.168	176.575	215.281	159.537	556.585	523.894	226.032	317.543	97.33978
	10	94.6216	123.869	95.3183	244.874	326.710	149.250	168.800	284.196	113.518	75.38247
Image 5	4	1525.07	1529.68	1529.63	2356.50	1356.78	1662.81	1220.39	1689.70	1558.81	776.9809
	6	623.770	650.887	636.915	634.497	469.579	1258.92	702.469	441.362	666.638	284.3285
	8	325.161	343.619	335.542	425.048	553.578	861.665	568.193	357.963	326.081	167.2593
	10	195.209	237.657	205.215	407.361	330.530	264.978	529.601	203.436	236.277	87.58311
Image 6	4	457.273	457.273	457.273	533.814	506.337	621.104	655.032	384.975	459.617	752.9573
	6	236.608	238.250	320.382	282.072	374.051	513.229	457.273	240.802	238.152	221.6207
	8	106.353	148.943	155.764	137.829	208.824	243.228	212.170	226.312	152.644	130.2358
	10	78.5562	109.097	79.6421	342.597	242.706	127.961	204.821	123.164	80.4963	94.10666
Image 7	4	185.090	185.090	185.090	248.551	795.662	229.447	737.426	802.177	185.090	392.2657
	6	69.0973	111.875	82.3258	159.289	173.142	680.915	98.5827	124.258	82.7000	176.0072
	8	28.1892	54.8811	55.5983	72.3988	88.4136	401.668	51.7245	123.975	30.2638	78.27992
	10	14.7190	65.3712	27.1964	59.3091	206.943	106.089	41.2723	78.3322	62.5614	63.22813
Image 8	4	354.124	363.613	363.613	931.783	817.366	532.874	537.243	363.320	363.613	582.895
	6	157.227	168.935	169.662	372.125	816.056	627.207	227.463	472.784	170.506	155.6182
	8	25.7542	88.5788	113.698	207.717	159.844	109.735	174.491	230.341	88.8945	89.52624
	10	62.0859	93.9654	60.9653	303.882	367.180	412.227	141.894	133.882	56.7455	49.61927
Image 9	4	677.871	690.033	690.033	1274.95	731.182	1211.21	767.929	729.868	679.526	479.4026
	6	354.950	360.679	356.051	435.445	544.529	857.653	406.786	475.113	356.846	248.325
	8	197.722	207.897	202.433	438.178	456.855	470.421	261.845	261.355	204.750	117.2682
	10	128.822	138.417	139.384	341.065	265.432	325.078	355.185	237.088	140.626	64.43033
Average		477.194	568.672	563.662	707.477	652.618	627.331	678.474	608.111	70.219	294.4718

**Table 4:** Comparison of SSIM computed by SAMFO-TH, MVO, WOA, FPA, SCA, ACO, PSO, ABC, and MFO with the proposed model using Kapur’s method with N = 4, 6, 8, and 10.

Image	N	SSIM-Kapur									
		SAMFO-TH	MVO	WOA	FPA	SCA	ACO	PSO	ABC	MFO	Proposed
Image 1	4	0.9275	0.9190	0.9209	0.9206	0.9016	0.8788	0.9193	0.9146	0.9213	0.9568
	6	0.9768	0.9600	0.9652	0.9624	0.9758	0.9419	0.9723	0.9681	0.9653	0.9713
	8	0.9882	0.9858	0.9830	0.9389	0.9860	0.9877	0.9837	0.9861	0.9852	0.9636
	10	0.9958	0.9947	0.9952	0.9879	0.9885	0.9927	0.9914	0.9949	0.9908	0.9932
Image 2	4	0.9386	0.9353	0.9339	0.9475	0.9295	0.9353	0.9305	0.9357	0.9357	0.9619
	6	0.9785	0.9735	0.9731	0.9634	0.9636	0.9662	0.9762	0.9738	0.9741	0.9815
	8	0.9935	0.9874	0.9861	0.9794	0.9878	0.9790	0.9896	0.9924	0.9920	0.9967
	10	0.9953	0.9951	0.9944	0.9850	0.9894	0.9907	0.9876	0.9934	0.9952	0.9971
Image 3	4	0.9417	0.9393	0.9395	0.9382	0.9404	0.9332	0.9397	0.9390	0.9397	0.961
	6	0.9611	0.9460	0.9464	0.9733	0.9549	0.9649	0.9559	0.9432	0.9490	0.9724
	8	0.9785	0.9673	0.9662	0.9572	0.9713	0.9615	0.9726	0.9766	0.9675	0.9881
	10	0.9890	0.9772	0.9725	0.9694	0.9853	0.9809	0.9722	0.9882	0.9770	0.9888
Image 4	4	0.9886	0.9881	0.9881	0.9873	0.9876	0.9878	0.9879	0.9885	0.9879	0.9988
	6	0.9942	0.9932	0.9934	0.9897	0.9905	0.9922	0.9873	0.9904	0.9939	0.9971
	8	0.9959	0.9951	0.9959	0.9938	0.9919	0.9877	0.9883	0.9934	0.9955	0.9921
	10	0.9973	0.9971	0.9972	0.9924	0.9910	0.9894	0.9951	0.9943	0.9970	0.999
Image 5	4	0.9699	0.9695	0.9695	0.9632	0.9638	0.9606	0.9695	0.9658	0.9691	0.9964
	6	0.9869	0.9858	0.9865	0.9817	0.9836	0.9789	0.9855	0.9894	0.9863	0.9921
	8	0.9916	0.9915	0.9914	0.9910	0.9872	0.9859	0.9833	0.9918	0.9911	0.9986
	10	0.9952	0.9949	0.9945	0.9936	0.9932	0.9920	0.9917	0.9953	0.9943	0.9934
Image 6	4	0.9887	0.9887	0.9887	0.9870	0.9837	0.9826	0.9766	0.9874	0.9888	0.9885
	6	0.9935	0.9927	0.9935	0.9899	0.9906	0.9885	0.9903	0.9930	0.9931	0.98871
	8	0.9965	0.9960	0.9957	0.9950	0.9918	0.9928	0.9934	0.9945	0.9959	0.9984
	10	0.9978	0.9973	0.9975	0.9935	0.9921	0.9932	0.9929	0.9968	0.9975	0.9986
Image 7	4	0.9915	0.9811	0.9811	0.9810	0.9768	0.9768	0.9730	0.9759	0.9809	0.9939
	6	0.9950	0.9946	0.9946	0.9838	0.9883	0.9903	0.9913	0.9931	0.9948	0.9252
	8	0.9971	0.9954	0.9959	0.9932	0.9952	0.9956	0.9888	0.9947	0.9958	0.9994
	10	0.9980	0.9977	0.9964	0.9972	0.9956	0.9948	0.9927	0.9973	0.9976	0.9908
Image 8	4	0.9855	0.9853	0.9853	0.9771	0.9797	0.9796	0.9799	0.9851	0.9853	0.9918
	6	0.9932	0.9930	0.9929	0.9900	0.9840	0.9820	0.9850	0.9899	0.9931	0.9899
	8	0.9961	0.9956	0.9954	0.9912	0.9939	0.9905	0.9936	0.9918	0.9954	0.9829
	10	0.9974	0.9969	0.9971	0.9934	0.9920	0.9909	0.9949	0.9946	0.9971	0.9995
Image 9	4	0.9799	0.9795	0.9795	0.9735	0.9774	0.9708	0.9744	0.9742	0.9796	0.993
	6	0.9902	0.9898	0.9895	0.9767	0.9805	0.9816	0.9857	0.9881	0.9892	0.9892
	8	0.9935	0.9934	0.9933	0.9843	0.9903	0.9895	0.9922	0.9933	0.9932	0.9976
	10	0.9963	0.9962	0.9961	0.9918	0.9855	0.9926	0.9920	0.9938	0.9962	0.9948
Average		0.98539	0.982472	0.98237	0.97811	0.97945	0.97720	0.97989	0.98217	0.98281	0.9867

**Table 5:** Comparison of FSIM computed by SAMFO-TH, MVO, WOA, FPA, SCA, ACO, PSO, ABC, and MFO with the proposed model using Kapur’s method with N = 4, 6, 8, and 10.

Image	N	FSIM									
		SAMFO-TH	MVO	WOA	FPA	SCA	ACO	PSO	ABC	MFO	Proposed
Image 1	4	0.6875	0.6716	0.6749	0.6783	0.6558	0.6183	0.6754	0.6725	0.6754	0.74
	6	0.7756	0.7626	0.7607	0.7405	0.7728	0.7182	0.7392	0.7532	0.7679	0.8089
	8	0.8377	0.8365	0.8217	0.7294	0.8117	0.7807	0.7907	0.8218	0.8319	0.8787
	10	0.8988	0.8890	0.8903	0.8268	0.8315	0.8444	0.8313	0.8727	0.8733	0.9108
Image	4	0.7613	0.7611	0.7597	0.7578	0.7452	0.7329	0.7496	0.7590	0.7563	0.7461
	6	0.8234	0.8114	0.8109	0.7829	0.7881	0.8038	0.8111	0.8077	0.8150	0.8696
	8	0.8982	0.8769	0.8704	0.8184	0.8409	0.8127	0.8499	0.8835	0.8910	0.8978
	10	0.9233	0.9206	0.9153	0.8541	0.8561	0.8782	0.8540	0.9007	0.9216	0.9421
Image	4	0.7674	0.7664	0.7662	0.7441	0.7665	0.7537	0.7469	0.7681	0.7670	0.8265
	6	0.8118	0.8001	0.7997	0.7927	0.7977	0.7878	0.7765	0.7995	0.8005	0.8310
	8	0.8344	0.8326	0.8324	0.7906	0.8188	0.8149	0.8230	0.8355	0.8330	0.9001
	10	0.8663	0.8561	0.8517	0.7883	0.8428	0.8417	0.8339	0.8691	0.8629	0.9168
Image 4	4	0.8245	0.8241	0.8242	0.8196	0.8161	0.8114	0.8149	0.8183	0.8205	0.7890
	6	0.8852	0.8756	0.8746	0.8334	0.8330	0.8650	0.8300	0.8391	0.8726	0.8751
	8	0.9122	0.9094	0.8972	0.8718	0.8583	0.8294	0.8176	0.8775	0.9003	0.9129
	10	0.9349	0.9246	0.9341	0.8766	0.8367	0.8171	0.8906	0.8766	0.9283	0.9446
Image 5	4	0.7856	0.7854	0.7854	0.7641	0.7842	0.7222	0.7585	0.7809	0.7842	0.7800
	6	0.8603	0.8576	0.8551	0.8370	0.8397	0.8069	0.8318	0.8678	0.8596	0.9032
	8	0.9010	0.8983	0.9007	0.8793	0.8568	0.8419	0.8550	0.9038	0.8993	0.9350
	10	0.9308	0.9287	0.9305	0.8953	0.9008	0.8995	0.8811	0.9311	0.9306	0.9834
Image 6	4	0.7456	0.7454	0.7454	0.7311	0.7209	0.7069	0.7327	0.7359	0.7453	0.7363
	6	0.8079	0.7953	0.8077	0.7717	0.7710	0.7751	0.7684	0.8032	0.7963	0.8682
	8	0.8682	0.8544	0.8662	0.8326	0.7871	0.8044	0.8282	0.8236	0.8659	0.8621
	10	0.8965	0.8862	0.8946	0.8066	0.7853	0.8316	0.8154	0.8731	0.8828	0.8851
Image 7	4	0.9068	0.8824	0.8792	0.8687	0.8546	0.8298	0.8384	0.8384	0.8774	0.9252
	6	0.9418	0.9377	0.9376	0.8928	0.8940	0.8857	0.8980	0.8921	0.9400	0.9198
	8	0.9623	0.9500	0.9537	0.9161	0.9393	0.9279	0.8830	0.9298	0.9569	0.9647
	10	0.9732	0.9707	0.9633	0.9553	0.9182	0.9146	0.9186	0.9442	0.9657	0.9746
Image 8	4	0.9160	0.9139	0.9139	0.8562	0.8902	0.8572	0.8179	0.9099	0.9139	0.9111
	6	0.9593	0.9591	0.9592	0.9170	0.9100	0.8888	0.9158	0.9004	0.9589	0.9599
	8	0.9716	0.9772	0.9740	0.9472	0.9555	0.9605	0.9378	0.9537	0.9736	0.9759
	10	0.9805	0.9849	0.9846	0.9479	0.9473	0.9299	0.9595	0.9620	0.9812	0.9827
Image 9	4	0.9290	0.9289	0.9286	0.8854	0.9117	0.8562	0.8693	0.9203	0.9282	0.8489
	6	0.9539	0.9577	0.9574	0.9050	0.9361	0.9106	0.9135	0.9472	0.9566	0.9541
	8	0.9622	0.9682	0.9672	0.9468	0.9437	0.9495	0.9489	0.9658	0.9676	0.9756
	10	0.9792	0.9797	0.9790	0.9558	0.9479	0.9613	0.9523	0.9630	0.9783	0.9897
Average		0.8798	0.8744	0.87409	0.83936	0.84350	0.83251	0.83774	0.86113	0.87443	0.89237

**Table 6:** Comparison of PRI computed by SAMFO-TH, MVO, WOA, FPA, SCA, ACO, PSO, ABC, and MFO with the proposed model using Kapur’s method with N = 4, 6, 8, and 10.

Image	N	PRI-Kapur-EMO									
		SAMFO-TH	MVO	WOA	FPA	SCA	ACO	PSO	ABC	MFO	Proposed
Image 1	4	0.6470	0.6352	0.6418	0.5833	0.5986	0.4005	0.6423	0.6350	0.6422	0.5264
	6	0.7467	0.7427	0.7182	0.7070	0.7198	0.6157	0.6552	0.6828	0.7247	0.6681
	8	0.7948	0.7945	0.7693	0.6684	0.7489	0.6820	0.7519	0.7934	0.7839	0.7899
	10	0.8282	0.82827	0.8201	0.7990	0.7611	0.7693	0.7777	0.8033	0.8170	0.8188
Image 2	4	0.6210	0.6008	0.5931	0.6372	0.5361	0.5523	0.5907	0.5731	0.6010	0.5698
	6	0.7332	0.7214	0.7234	0.7045	0.7064	0.7020	0.7147	0.7121	0.6969	0.6712
	8	0.7919	0.7797	0.7790	0.7006	0.7050	0.7393	0.7059	0.7802	0.7729	0.7458
	10	0.8265	0.8078	0.8013	0.7267	0.7053	0.8069	0.7511	0.7678	0.8250	0.8225
Image 3	4	0.5290	0.5092	0.5109	0.5044	0.5226	0.4611	0.5122	0.5103	0.5125	0.4985
	6	0.6597	0.5466	0.5496	0.6219	0.6039	0.6781	0.6335	0.5237	0.5700	0.63255
	8	0.7125	0.6771	0.6647	0.6287	0.6602	0.6378	0.7067	0.7190	0.6807	0.6043
	10	0.7903	0.7526	0.7165	0.6949	0.7875	0.7894	0.6729	0.7781	0.7496	0.7192
Image 4	4	0.6152	0.6193	0.6193	0.6214	0.6046	0.6412	0.6049	0.6004	0.6117	0.4589
	6	0.7180	0.7014	0.6903	0.6401	0.6035	0.6577	0.6116	0.6421	0.6935	0.6546
	8	0.7476	0.7558	0.7605	0.7389	0.6795	0.7027	0.6658	0.7412	0.7263	0.721
	10	0.7966	0.7927	0.7802	0.7521	0.6231	0.7273	0.7363	0.7132	0.7840	0.7524
Image 5	4	0.7942	0.7914	0.7925	0.7704	0.7677	0.7311	0.7576	0.7902	0.7939	0.7542
	6	0.8487	0.8364	0.8441	0.8348	0.8201	0.7911	0.8087	0.8466	0.8433	0.8525
	8	0.8838	0.8815	0.8830	0.8595	0.8463	0.8234	0.8523	0.8750	0.8822	0.8778
	10	0.9048	0.9031	0.9057	0.8831	0.8706	0.8735	0.8673	0.8951	0.9003	0.8952
Image 6	4	0.6642	0.6639	0.6642	0.6286	0.6329	0.5797	0.6621	0.6638	0.6634	0.61258
	6	0.7566	0.7414	0.7453	0.7465	0.6773	0.7694	0.6809	0.7580	0.7231	0.7299
	8	0.8141	0.7982	0.7906	0.7986	0.7316	0.7832	0.7763	0.7762	0.7948	0.7788
	10	0.8455	0.8276	0.8431	0.7847	0.7402	0.7664	0.7938	0.8281	0.8320	0.8436
Image 7	4	0.4376	0.3253	0.3242	0.3180	0.3403	0.3340	0.2412	0.2772	0.3280	0.3658
	6	0.4615	0.4502	0.4540	0.4148	0.3937	0.3654	0.3925	0.4566	0.4581	0.4589
	8	0.5227	0.5154	0.4970	0.5117	0.4590	0.4837	0.4901	0.4265	0.5133	0.6384
	10	0.6222	0.5989	0.5442	0.5579	0.5417	0.6113	0.5790	0.5440	0.5320	0.6683
Image 8	4	0.7585	0.7337	0.7337	0.7337	0.7143	0.7049	0.7005	0.7183	0.7341	0.6867
	6	0.8129	0.8096	0.8094	0.7717	0.7809	0.7490	0.7476	0.7663	0.8017	0.8423
	8	0.8544	0.8464	0.8360	0.7986	0.8283	0.7916	0.8389	0.7878	0.8395	0.8507
	10	0.8738	0.8612	0.8717	0.8397	0.7780	0.7937	0.8621	0.8378	0.8684	0.865
Image 9	4	0.7531	0.7520	0.7514	0.7140	0.7380	0.6981	0.7412	0.7518	0.7524	0.6142
	6	0.8214	0.8201	0.8041	0.7613	0.7619	0.7749	0.7381	0.7831	0.8200	0.6983
	8	0.8563	0.8479	0.8494	0.8191	0.8003	0.8017	0.8063	0.8350	0.8337	0.775
	10	0.8825	0.8765	0.8760	0.8490	0.8210	0.8411	0.8425	0.8437	0.8726	0.8384
Average		0.7424	0.7262	0.7210	0.6979	0.6836	0.6841	0.6920	0.7065	0.7216	0.7027

**Table 7:** Comparison of VOI computed by SAMFO-TH, MVO, WOA, FPA, SCA, ACO, PSO, ABC, and MFO with the proposed model using Kapur’s method with N = 4, 6, 8, and 10.

Image	N	VOI-Kapur-EMO									
		SAMFO-TH	MVO	WOA	FPA	SCA	ACO	PSO	ABC	MFO	Proposed
Image 1	4	5.3330	5.3576	5.3336	5.4371	5.4764	5.9613	5.2945	5.3470	5.3324	5.4929
	6	4.8227	4.8497	4.9617	4.9534	4.9410	5.2220	5.1722	5.0549	4.9153	4.7986
	8	4.5099	4.5170	4.6569	5.0336	4.7645	4.9313	4.7757	4.5355	4.5731	4.4826
	10	4.2423	4.3121	4.3065	4.4640	4.6410	4.5176	4.5288	4.3774	4.3215	4.2165
Image 2	4	5.2284	5.3003	5.3176	5.2022	5.4836	5.4641	5.3048	5.3813	5.2958	5.1645
	6	4.7333	4.8488	4.8459	4.9018	4.9639	4.8143	4.9237	4.8548	4.7964	4.8883
	8	4.4419	4.5026	4.5222	4.8948	4.9212	4.9252	4.7919	4.5447	4.5486	4.3051
	10	4.2167	4.2874	4.3251	4.5219	4.7620	4.3481	4.6797	4.4438	4.2172	4.1258
Image 3	4	5.1486	5.1894	5.1876	5.2404	5.1837	5.3184	5.1667	5.1845	5.1814	5.1224
	6	4.6392	4.9628	4.9574	4.6476	4.8242	4.7506	4.7276	5.0135	4.9010	4.6039
	8	4.4126	4.5210	4.5607	4.7344	4.6110	4.6030	4.4475	4.3978	4.5123	4.3412
	10	4.1030	4.1800	4.3102	4.4259	4.1170	4.1168	4.4745	4.1607	4.1549	4.1243
Image 4	4	5.1787	5.1617	5.1614	5.1402	5.2015	5.0637	5.1667	5.1457	5.1851	5.1776
	6	4.7021	4.7650	4.8077	4.9795	5.0493	4.7123	4.9176	4.9828	4.8211	4.6573
	8	4.4405	4.4753	4.4438	4.5795	4.7648	4.6895	4.8842	4.5344	4.5506	4.3403
	10	4.2049	4.2094	4.2883	4.4242	4.8911	4.6964	4.4474	4.5626	4.2779	4.2658
Image 5	4	5.0841	5.0933	5.0841	5.1615	5.2042	5.3147	5.2377	5.1676	5.0848	5.0065
	6	4.6610	4.7338	4.6999	4.7572	4.8916	4.9611	4.8981	4.6937	4.7025	4.6558
	8	4.3212	4.3385	4.3278	4.4909	4.6591	4.6347	4.5010	4.3848	4.3361	4.2331
	10	4.0469	4.0638	4.1039	4.2530	4.4220	4.3279	4.3399	4.1630	4.0583	4.3297
Image 6	4	5.2650	5.2676	5.2650	5.3632	5.3958	5.5519	5.2872	5.2788	5.2674	5.1710
	6	4.7954	4.9082	4.8970	4.8278	5.1177	4.9170	5.0589	4.7993	4.9751	4.4071
	8	4.4488	4.5450	4.6060	4.5046	4.9017	4.5562	4.6108	4.6814	4.5639	4.0305
	10	4.1773	4.3155	4.2042	4.4149	4.8285	4.5117	4.5394	4.2892	4.2814	4.3887
Image 7	4	4.4371	4.6715	4.6934	4.6916	4.6462	4.7513	4.9261	4.8303	4.6838	4.7709
	6	4.3105	4.3017	4.3222	4.3926	4.5140	4.5557	4.4991	4.3334	4.3303	4.4257
	8	4.0766	4.0629	4.1389	3.8737	4.2378	4.1750	4.2134	4.3480	4.1253	4.0134
	10	3.7302	3.8034	3.9591	3.8714	3.9576	3.7968	3.9279	3.9594	3.9441	3.5823
Image 8	4	5.2353	5.2396	5.2396	5.3274	5.3096	5.2584	5.3555	5.2864	5.2396	5.1201
	6	4.7303	4.7425	4.7413	4.8706	4.8987	5.0754	5.0003	4.9568	4.7932	4.7156
	8	4.3851	4.4530	4.5217	4.7100	4.6106	4.6958	4.4705	4.7702	4.5139	4.7666
	10	4.1701	4.2839	4.1797	4.3947	4.7208	4.6088	4.2242	4.4325	4.2204	4.2265
Image 9	4	5.2527	5.2685	5.2758	5.3564	5.3178	5.4439	5.2739	5.3524	5.2705	5.1827
	6	4.8043	4.8097	4.8980	5.0513	5.1684	4.9999	5.1457	5.0007	4.8105	4.7817
	8	4.4959	4.5616	4.5543	4.7000	4.8973	4.8750	4.8079	4.6274	4.6573	4.4210
	10	4.2281	4.2806	4.2873	4.4128	4.7177	4.4756	4.4871	4.4928	4.3162	4.1244
Average		4.5837	4.6440	4.6662	4.75016	4.8614	4.82281	4.79189	4.73248	4.65997	4.5683

**Table 8:** Comparison of MEAN computed by SAMFO-TH, MVO, WOA, FPA, SCA, ACO, PSO, ABC, and MFOwith the proposed model using Kapur’s method with N = 4, 6, 8, and 10.

		Proposed Method					
	N	EMO_Kapur			EMO_OTSU		
Image		R	G	B	R	G	B
Image 1	4	22.7245	20.6617	21.9151	1.8715 × 10^10^	2.7645 × 10^10^	2.3448 × 10^10^
	6	31.3838	31.1731	30.2143	1.8715 × 10^10^	2.7645 × 10^10^	2.3448 × 10^10^
	8	38.9241	37.9386	37.6682	1.8715 × 10^10^	2.7645 × 10^10^	2.3448 × 10^10^
	10	45.6957	44.2571	44.3222	1.8715 × 10^10^	2.7645 × 10^10^	2.3448 × 10^10^
Image 2	4	23.1335	21.3695	21.3695	1.2788 × 10^11^	2.2774 × 10^11^	2.2464 × 10^11^
	6	31.7350	30.2454	30.6368	2.2774 × 10^11^	1.2788 × 10^11^	2.2464 × 10^11^
	8	38.7069	37.1543	37.7688	2.2774 × 10^11^	2.2464 × 10^11^	1.2788 × 10^11^
	10	45.7586	45.4235	44.4192	2.2774 × 10^11^	2.2464 × 10^11^	1.2788 × 10^11^
Image 3	4	23.3813	20.7151	23.1100	1.3372 × 10^10^	1.0266 × 10^10^	8.4907 × 10^9^
	6	31.6050	30.3837	31.5643	1.3372 × 10^10^	1.0266 × 10^10^	8.4907 × 10^9^
	8	39.2754	38.5151	39.1151	1.3372 × 10^10^	1.0266 × 10^10^	8.4907 × 10^9^
	10	46.0921	45.6209	44.8967	1.3372 × 10^10^	1.0266 × 10^10^	8.4907 × 10^9^
Image 4	4	22.0506	20.3480	22.4926	7.1205 × 10^10^	1.1270 × 10^11^	6.8970 × 10^10^
	6	30.1096	28.7478	29.7487	7.1205 × 10^10^	1.1270 × 10^11^	6.8970 × 10^10^
	8	37.5105	36.9517	38.4094	7.1205 × 10^10^	1.1270 × 10^11^	6.8970 × 10^10^
	10	44.1471	43.7316	44.8359	7.1205 × 10^10^	1.1270 × 10^11^	6.8970 × 10^10^
Image 5	4	23.0147	21.6803	22.5429	2.8927 × 10^11^	3.1468 × 10^11^	2.4490 × 10^11^
	6	31.1646	31.0380	30.4736	2.8927 × 10^11^	3.1468 × 10^11^	2.4490 × 10^11^
	8	38.3985	37.7448	38.5851	2.8927 × 10^11^	3.1468 × 10^11^	2.4490 × 10^11^
	10	44.9580	45.6851	45.2806	2.8927 × 10^11^	3.1468 × 10^11^	2.4490 × 10^11^
Image 6	4	22.4669	21.5328	21.5243	2.8820 × 10^10^	2.3478 × 10^10^	8.8752 × 10^9^
	6	31.3892	29.5077	29.6574	2.8820 × 10^10^	2.3478 × 10^10^	8.8752 × 10^9^
	8	39.0856	37.1529	37.6348	2.8820 × 10^10^	2.3478 × 10^10^	8.8752 × 10^9^
	10	45.9102	42.9632	44.0875	2.8820 × 10^10^	2.3478 × 10^10^	8.8752 × 10^9^
Image 7	4	22.9682	19.4795	21.3255	1.8752 × 10^10^	1.6944 × 10^10^	3.6049 × 10^9^
	6	31.2527	29.0121	30.4313	1.8752 × 10^10^	1.6944 × 10^10^	3.6049 × 10^9^
	8	38.2344	35.3177	37.6600	1.8752 × 10^10^	1.6944 × 10^10^	3.6049 × 10^9^
	10	45.5786	42.0219	43.9885	1.8752 × 10^10^	1.6944 × 10^10^	3.6049 × 10^9^
Image 8	4	22.5804	22.6632	21.8942	1.1475 × 10^11^	1.0766 × 10^11^	5.5300 × 10^10^
	6	35.5656	33.8955	34.6598	1.1475 × 10^11^	1.0766 × 10^11^	5.5300 × 10^10^
	8	37.9865	37.9041	38.1715	1.1475 × 10^11^	1.0766 × 10^11^	5.5300 × 10^10^
	10	45.7055	44.2770	45.0063	1.1475 × 10^11^	1.0766 × 10^11^	5.5300 × 10^10^
Image 9	4	79.7932	79.2440	79.2472	2.1651 × 10^11^	3.3482 × 10^11^	3.7903 × 10^11^
	6	22.3258	22.0532	21.7375	2.1651 × 10^11^	3.3482 × 10^11^	3.7903 × 10^11^
	8	31.4954	29.6734	30.1372	2.1651 × 10^11^	3.3482 × 10^11^	3.7903 × 10^11^
	10	39.0669	37.7023	37.6457	2.1651 × 10^11^	3.3482 × 10^11^	3.7903 × 10^11^
Average		42.72888	41.809	41.9909	1.83× 10^11^	2.59× 10^11^	2.71× 10^11^

**Table 9:** Comparison of PSNR computed by SAMFO-TH, MVO, WOA, FPA, SCA, ACO, PSO, ABC, and MFOwith the proposed model using Otsu’s method with N = 4, 6, 8, and 10.

Image	N	PSNR									
		SAMFO-TH	MVO	WOA	FPA	SCA	ACO	PSO	ABC	MFO	Proposed
Image 1	4	16.3860	16.4406	16.4308	16.1198	15.9518	16.5188	15.9471	15.7504	16.3509	19.6804
	6	18.9503	18.6464	18.6453	17.0514	18.8871	16.2863	17.6779	18.8405	18.6600	22.3901
	8	22.1062	21.6261	23.1484	19.1913	20.1582	24.3537	18.3762	22.1011	21.6192	23.9987
	10	23.7705	23.3273	23.3233	23.5426	22.8207	22.5577	22.1879	22.6202	22.8891	28.0707
Image 2	4	16.6237	16.8918	16.7502	15.5177	16.8216	15.2934	17.3576	16.6367	16.6237	18.8124
	6	19.9297	19.9122	20.1880	18.9867	21.2966	18.3149	20.8387	18.9461	19.9028	24.6275
	8	22.3808	22.2341	22.1322	24.7995	23.1557	21.8098	20.5504	22.4373	22.2379	24.8692
	10	25.5448	22.2341	24.6603	26.0898	25.3696	25.8321	20.5533	23.6078	24.6386	26.0680
Image 3	4	16.4184	16.1060	16.4101	13.9539	17.0046	18.1521	16.7390	16.3032	16.1370	20.5265
	6	19.9828	19.5770	19.6195	19.1009	15.4523	21.3334	19.6198	19.5441	19.5530	24.4963
	8	22.7395	22.0050	21.9373	19.5140	14.9593	21.9748	20.7408	16.1947	22.1665	28.0946
	10	24.2109	23.6942	23.6708	20.8159	23.3595	21.9945	26.2623	19.0748	23.4534	27.7046
Image 4	4	21.7144	21.7140	21.7517	21.2717	21.7111	23.5486	17.4448	21.6892	21.7140	26.2798
	6	24.9365	24.9499	24.9711	23.8558	23.2487	22.7919	23.2571	24.5169	24.9234	29.6004
	8	28.2781	27.8412	27.8172	25.0977	26.4994	26.4631	23.0717	26.3194	27.4682	32.1619
	10	29.7479	29.7163	28.8792	26.5819	19.5857	26.4818	28.1911	27.0843	29.3546	34.1414
Image 5	4	14.8363	14.8311	14.8393	12.3275	14.9024	13.5501	13.3716	14.3507	14.6782	19.5873
	6	17.7317	17.6955	17.5783	16.3833	16.4033	19.7073	17.9584	20.1421	17.7269	22.7009
	8	20.2708	20.0328	20.0432	19.2313	21.8799	20.3893	21.8624	20.5749	19.7951	24.5320
	10	21.9833	22.3498	22.9532	20.2590	21.6939	18.9397	22.8995	19.1012	21.2030	26.2652
Image 6	4	17.3196	17.4019	17.3845	16.6688	8.1594	12.7024	13.8483	14.6232	17.3196	23.3272
	6	22.0303	21.7427	21.3055	21.3326	8.3820	17.8780	23.8202	21.1869	21.4152	23.5649
	8	24.6298	23.5592	24.2151	23.9717	15.8327	24.4255	24.3540	10.3526	24.2388	27.4039
	10	27.6155	30.0282	26.7338	28.1801	15.3929	26.2118	26.7839	15.1460	26.9620	30.1257
Image 7	4	17.0406	17.0447	17.0409	15.6237	18.9268	26.6051	26.9731	16.7555	17.0104	26.8075
	6	19.3985	2.7706	2.7706	17.7746	16.3519	23.1112	22.4328	13.5358	19.3658	28.9907
	8	22.7231	2.7706	20.7583	27.0120	23.5447	29.1942	21.3682	22.1778	21.9322	32.2855
	10	27.8591	2.7706	22.6694	2.7706	28.7546	30.0138	12.3543	25.4857	2.8175	28.8837
Image 8	4	12.3553	12.4126	12.3557	12.4771	14.3196	10.9526	15.7630	19.3305	12.0893	21.3351
	6	17.8286	17.8180	17.8605	18.3484	17.9321	16.5747	21.3367	18.1996	17.7560	26.3956
	8	21.2291	3.4714	20.4676	20.4749	18.4503	20.7354	21.3140	21.6457	21.1268	28.9553
	10	24.0933	3.4714	22.6721	23.4736	22.7114	16.4386	24.3220	16.6142	23.8562	31.0910
Image 9	4	15.8561	15.6938	15.8340	14.9129	15.7650	14.7494	15.1135	15.7597	15.6550	21.0972
	6	19.4060	19.0694	18.9712	19.2740	16.0860	16.5833	18.6378	21.2941	18.8624	26.3328
	8	22.9089	22.4093	21.9344	20.4690	19.4735	20.6358	22.6642	23.2027	21.8292	30.3863
	10	25.2257	24.1457	24.6802	22.1094	22.3322	21.0518	22.7531	25.7185	23.7436	32.6120
Average		21.2795	18.289	20.3723	19.5712	18.9882	20.6710	20.5207	19.6351	20.1965	26.2278

**Table 10:** Comparison of MSE computed by SAMFO-TH, MVO, WOA, FPA, SCA, ACO, PSO, ABC, and MFO with the proposed model using Otsu’s method with N = 4, 6, 8, and 10.

Image	N	MSE									
		SAMFO-TH	MVO	WOA	FPA	SCA	ACO	PSO	ABC	MFO	Proposed
Image 1	4	1494.51	1475.82	1479.16	1588.94	1651.68	1449.46	1653.48	1730.05	1506.67	699.907
	6	828.028	888.056	888.275	1282.26	840.169	1529.14	1109.97	874.585	885.276	375.0338
	8	400.368	447.168	314.952	783.336	622.564	238.619	945.053	400.843	447.880	258.9467
	10	272.918	302.235	302.520	287.620	339.636	360.835	392.904	355.687	334.323	101.3936
Image 2	4	1414.80	1330.12	1374.24	1825.26	1351.81	1922.02	1194.93	1410.62	1414.80	854.7525
	6	660.860	663.536	622.700	821.135	482.416	958.506	536.061	828.859	664.974	224.0425
	8	375.835	388.752	397.975	215.342	314.418	428.643	572.847	354.681	388.413	211.9143
	10	181.383	388.752	222.356	159.992	188.852	169.775	572.463	283.335	223.471	160.7979
Image 3	4	1483.35	1594.03	1486.21	2616.30	1296.09	995.099	1377.87	1523.20	1582.63	576.0104
	6	652.823	716.768	709.797	799.811	1825.94	478.348	709.746	722.213	720.747	230.9141
	8	346.045	409.811	416.244	727.240	2075.66	412.666	548.278	1561.85	394.849	100.8371
	10	246.600	277.754	279.254	538.876	300.003	410.796	153.761	804.646	293.589	110.3114
Image 4	4	438.168	438.205	434.423	485.192	438.496	287.221	1171.15	440.713	438.205	153.1441
	6	208.656	208.012	206.998	267.610	307.761	341.890	307.164	229.82	209.286	71.29193
	8	96.6658	106.895	107.489	201.050	145.593	146.814	320.563	151.750	116.483	39.52668
	10	68.9119	69.4147	84.1704	142.853	715.340	146.185	98.6201	127.241	75.4424	25.05765
Image 5	4	2135.33	2137.82	2133.85	3840.84	2103.04	2871.26	2991.75	2387.92	2214.41	715.073
	6	1096.23	1105.40	1135.72	1495.41	1488.58	695.577	1040.57	629.324	1097.59	349.1326
	8	610.943	645.363	643.810	776.151	421.786	594.500	423.484	569.622	681.670	229.0237
	10	411.860	378.529	329.429	612.604	440.239	830.066	333.524	799.767	492.920	153.6598
Image 6	4	1205.40	1182.73	1187.55	1400.21	9934.32	3490.10	2680.7	2242.71	1205.47	302.2459
	6	407.424	435.319	481.429	478.427	9438.04	1059.96	269.818	494.758	469.419	286.1478
	8	223.925	286.523	246.359	260.561	1697.58	234.709	238.606	5995.41	245.019	118.2198
	10	112.598	64.6047	137.942	98.8710	1878.42	155.562	136.361	1988.32	130.882	63.16992
Image 7	4	1285.30	1284.14	1285.35	1781.25	832.535	142.093	130.548	1372.62	1291.32	135.6221
	6	746.842	3435.82	3435.82	1085.56	1506.27	317.659	371.367	2880.74	753.742	82.03711
	8	350.520	3435.82	546.068	129.383	287.479	78.2817	474.529	380.256	21.9322	38.41761
	10	105.163	3435.82	351.671	3435.82	86.6203	64.8187	3781.45	183.868	3398.81	84.08342
Image 8	4	3780.54	3731.0	3780.28	3676.02	2405.19	5221.88	1725.08	758.633	3786.56	478.1568
	6	1072.16	1074.7	1064.25	951.136	1046.80	1430.92	477.979	984.288	1080.15	149.1147
	8	489.974	2923.71	583.880	582.888	929.082	548.963	480.488	445.158	489.974	82.70854
	10	253.366	2923.71	351.453	292.227	348.290	1476.44	240.372	1418.05	253.366	50.58018
Image 9	4	1688.44	1752.75	1697.01	2097.92	1724.25	2178.42	1591.21	1762.36	1768.52	505.0802
	6	745.555	805.642	824.069	769.197	1601.33	1428.13	889.810	482.687	844.971	151.2866
	8	332.804	373.378	416.522	583.683	734.065	561.690	352.099	311.036	426.741	59.49088
	10	195.214	250.330	221.338	400.074	380.069	510.392	344.961	174.271	274.613	35.6353
Average		733.8752	1149.1232	838.3489	1041.4180	1449.4559	949.0955	851.0990	1057.2747	850.6977	229.5213

**Table 11:** Comparison of SSIM computed by SAMFO-TH, MVO, WOA, FPA, SCA, ACO, PSO, ABC, and MFO with the proposed model using Otsu’s method with N = 4, 6, 8, and 10.

Image	N	SSIM-O									
		SAMFO-TH	MVO	WOA	FPA	SCA	ACO	PSO	ABC	MFO	Proposed
Image 1	4	0.9394	0.9395	0.9395	0.9239	0.9361	0.9284	0.9365	0.9330	0.9393	0.8898
	6	0.9696	0.9669	0.9661	0.9486	0.9568	0.9624	0.9663	0.9672	0.9688	0.9755
	8	0.9847	0.9835	0.9843	0.9836	0.9822	0.9668	0.9784	0.9728	0.9817	0.9857
	10	0.9928	0.9883	0.9878	0.9898	0.9859	0.9882	0.9865	0.9877	0.9874	0.9954
Image 2	4	0.9472	0.9462	0.9469	0.9322	0.9311	0.9373	0.9465	0.9105	0.9466	0.9358
	6	0.9738	0.9737	0.9733	0.9669	0.9707	0.9677	0.9818	0.9569	0.9744	0.9748
	8	0.9844	0.9837	0.9836	0.9801	0.9779	0.9823	0.9825	0.9879	0.9837	0.9844
	10	0.9912	0.9901	0.9893	0.9873	0.9849	0.9898	0.9865	0.9910	0.9910	0.9955
Image 3	4	0.9559	0.9461	0.9469	0.9289	0.9463	0.9430	0.9458	0.9474	0.9481	0.9599
	6	0.9781	0.9766	0.9767	0.9700	0.9550	0.9728	0.9771	0.9744	0.9764	0.9689
	8	0.9871	0.9856	0.9852	0.9843	0.9642	0.9865	0.9776	0.9707	0.9865	0.9879
	10	0.9907	0.9893	0.9890	0.9832	0.9897	0.9744	0.9890	0.9748	0.9890	0.9911
Image 4	4	0.9857	0.9857	0.9858	0.9839	0.9860	0.9774	0.9780	0.9859	0.9858	0.9861
	6	0.9938	0.9937	0.9935	0.9906	0.9919	0.9907	0.9925	0.9937	0.9938	0.9942
	8	0.9968	0.9967	0.9967	0.9948	0.9945	0.9939	0.9936	0.9958	0.9967	0.9971
	10	0.9978	0.9976	0.9974	0.9950	0.9838	0.9958	0.9966	0.9967	0.9977	0.9979
Image 5	4	0.9416	0.9408	0.9414	0.9329	0.9453	0.9314	0.9390	0.9283	0.9410	0.95187
	6	0.9783	0.9700	0.9694	0.9702	0.9602	0.9723	0.9750	0.9709	0.9697	0.98109
	8	0.9865	0.9825	0.9828	0.9792	0.9826	0.9798	0.9837	0.9854	0.9826	0.9877
	10	0.9892	0.9887	0.9889	0.9814	0.9839	0.9799	0.9890	0.9832	0.9877	0.9900
Image 6	4	0.9540	0.9539	0.9542	0.9258	0.8634	0.9504	0.9223	0.9553	0.9538	0.9489
	6	0.9842	0.9795	0.9792	0.9784	0.9062	0.9427	0.9817	0.9786	0.9821	0.9852
	8	0.9913	0.9911	0.9910	0.9800	0.9646	0.9874	0.9908	0.9264	0.9914	0.9925
	10	0.9975	0.9968	0.9958	0.9946	0.9696	0.9928	0.9935	0.9760	0.9959	0.9988
Image 7	4	0.9887	0.9805	0.9805	0.9611	0.9866	0.9802	0.9796	0.9865	0.9802	0.98898
	6	0.9890	0.7963	0.7964	0.9910	0.9811	0.9939	0.9827	0.9724	0.9890	0.9945
	8	0.9953	0.7987	0.9923	0.9928	0.9946	0.9936	0.9926	0.9810	0.9936	0.9968
	10	0.9969	0.6464	0.9949	0.7993	0.9811	0.9958	0.9794	0.9950	0.8029	0.9978
Image 8	4	0.9371	0.9371	0.9372	0.9656	0.9560	0.9305	0.9351	0.9226	0.9369	0.9487
	6	0.9842	0.9804	0.9804	0.9802	0.9787	0.9692	0.9808	0.9855	0.9806	0.9845
	8	0.9912	0.8135	0.9899	0.9597	0.9875	0.9910	0.9886	0.9903	0.9906	0.9925
	10	0.9952	0.8156	0.9939	0.9860	0.9921	0.9874	0.9935	0.9859	0.9949	0.9958
Image 9	4	0.9517	0.9511	0.9511	0.9477	0.9508	0.9292	0.9472	0.9506	0.9514	0.9625
	6	0.9810	0.9793	0.9789	0.9674	0.9639	0.9740	0.9672	0.9807	0.9791	0.9811
	8	0.9894	0.9869	0.9887	0.9855	0.9776	0.9860	0.9818	0.9870	0.9870	0.9985
	10	0.9952	0.9947	0.9941	0.9904	0.9863	0.9905	0.9901	0.9958	0.9939	0.8898
Average		0.9801	0.9479	0.9728	0.9670	0.9680	0.972	0.9752	0.9717	0.9730	0.9803

**Table 12:** Comparison of FSIM computed by SAMFO-TH, MVO, WOA, FPA, SCA, ACO, PSO, ABC, and MFO with the proposed model using Otsu’s method with N = 4, 6, 8, and 10.

Image	N	FSIM									
		SAMFO-TH	MVO	WOA	FPA	SCA	ACO	PSO	ABC	MFO	Proposed
Image 1	4	0.6964	0.6988	0.6991	0.6818	0.6945	0.6882	0.6789	0.6468	0.6960	0.7303
	6	0.7898	0.7800	0.7772	0.7372	0.7239	0.7316	0.7779	0.7855	0.7782	0.8091
	8	0.8357	0.8328	0.8332	0.8245	0.7911	0.7670	0.8018	0.7181	0.8299	0.8446
	10	0.8705	0.8704	0.8688	0.8431	0.7797	0.8132	0.8512	0.7951	0.8667	0.8934
Image 2	4	0.7564	0.7457	0.7544	0.7359	0.6661	0.7326	0.7440	0.7039	0.7525	0.8099
	6	0.8201	0.8165	0.8198	0.7929	0.7701	0.8025	0.8082	0.7514	0.8174	0.8706
	8	0.8655	0.8633	0.8609	0.8785	0.8118	0.8241	0.8505	0.8414	0.8592	0.8579
	10	0.9022	0.8977	0.8950	0.8792	0.9014	0.8788	0.8615	0.8758	0.9003	0.8443
Image 3	4	0.7309	0.7303	0.7368	0.7451	0.7412	0.7112	0.7245	0.7359	0.7312	0.8224
	6	0.7973	0.7915	0.7951	0.7713	0.7622	0.7756	0.7912	0.7895	0.7922	0.8786
	8	0.8494	0.8422	0.8427	0.8236	0.7705	0.8079	0.8035	0.8034	0.8337	0.9181
	10	0.8762	0.8692	0.8690	0.8311	0.8409	0.8021	0.8562	0.8070	0.8728	0.9189
Image 4	4	0.8214	0.8212	0.8212	0.8112	0.8205	0.7817	0.7841	0.8215	0.8212	0.8430
	6	0.8910	0.8914	0.8941	0.8700	0.8675	0.8462	0.8712	0.8852	0.8926	0.9124
	8	0.9318	0.9305	0.9315	0.9019	0.9031	0.8909	0.8775	0.9170	0.9286	0.9318
	10	0.9500	0.9483	0.9464	0.9035	0.8112	0.9184	0.9271	0.9269	0.9479	0.9446
Image 5	4	0.7487	0.7450	0.7474	0.7123	0.6931	0.7053	0.7260	0.6547	0.7471	0.8122
	6	0.8329	0.8298	0.8327	0.8114	0.7323	0.7935	0.8148	0.8340	0.8325	0.8663
	8	0.8788	0.8773	0.8734	0.8670	0.8156	0.8438	0.8676	0.8677	0.8748	0.8961
	10	0.9027	0.9017	0.9061	0.8491	0.8379	0.8548	0.8870	0.8569	0.8992	0.9160
Image 6	4	0.7146	0.7118	0.7134	0.6894	0.6311	0.6550	0.6651	0.6936	0.7103	0.7740
	6	0.8061	0.8016	0.7956	0.7954	0.7113	0.6985	0.7667	0.7508	0.8000	0.7830
	8	0.8567	0.8529	0.8540	0.8085	0.6770	0.7953	0.8009	0.7403	0.8560	0.8376
	10	0.9117	0.8988	0.8993	0.8354	0.7020	0.8283	0.8384	0.7161	0.8989	0.8710
Image 7	4	0.8415	0.8435	0.8439	0.7543	0.8313	0.8670	0.8120	0.8616	0.8383	0.8982
	6	0.9093	NaN	NaN	0.8888	0.8326	0.8953	0.8560	NaN	0.9075	0.9455
	8	0.9369	NaN	0.9311	0.8700	0.9038	0.9187	0.8522	0.8636	0.9367	0.9614
	10	0.9559	NaN	0.9486	NaN	0.8531	0.9307	0.8982	0.8894	NaN	0.9462
Image 8	4	0.8125	0.8173	0.8138	0.8777	0.7630	0.7839	0.8035	0.8073	0.8111	0.8636
	6	0.8968	0.8958	0.8965	0.9127	0.8890	0.8600	0.8928	0.8789	0.8962	0.9294
	8	0.9379	NaN	0.9343	0.8637	0.8632	0.9329	0.9164	0.9310	0.9346	0.9545
	10	0.9594	NaN	0.9561	0.9124	0.9400	0.8680	0.9538	0.9166	0.9574	0.9683
Image 9	4	0.8632	0.8501	0.8511	0.8476	0.8478	0.8133	0.8411	0.8515	0.8562	0.8645
	6	0.9275	0.9219	0.9179	0.8992	0.8840	0.8915	0.8827	0.9238	0.9263	0.9125
	8	0.9491	0.9440	0.9495	0.9318	0.8575	0.9209	0.9225	0.9437	0.9421	0.9375
	10	0.9681	0.9653	0.9652	0.9572	0.9199	0.9544	0.9407	0.9757	0.9628	0.9775
Average		0.8609	0.844	0.8564	0.8318	0.8011	0.8217	0.8318	0.8217	0.8545	0.8818

**Table 13:** Comparison computed by SAMFO-TH, MVO, WOA, FPA, SCA, ACO, PSO, ABC, and MFO with the proposed model using Otsu’s method with N = 4, 6, 8, and 10.

Image	N	PRI for Otsu with EMO									
		SAMFO-TH	MVO	WOA	FPA	SCA	ACO	PSO	ABC	MFO	Proposed
Image 1	4	0.6945	0.6941	0.6944	0.6502	0.6877	0.6805	0.6783	0.5500	0.6943	0.6854
	6	0.8013	0.7993	0.7976	0.7546	0.7143	0.6889	0.7569	0.8008	0.7996	0.8099
	8	0.8483	0.8471	0.8385	0.8253	0.7470	0.7660	0.7971	0.5964	0.8451	0.8486
	10	0.8871	0.8810	0.8799	0.8345	0.6986	0.7743	0.8192	0.7571	0.8792	0.8945
Image 2	4	0.6603	0.6598	0.6599	0.6220	0.3651	0.5653	0.5918	0.5077	0.6597	0.6877
	6	0.7905	0.7898	0.7894	0.7473	0.6506	0.7530	0.6818	0.6818	0.7875	0.7819
	8	0.8410	0.8415	0.8429	0.7968	0.7797	0.7674	0.7819	0.7491	0.8404	0.8519
	10	0.8755	0.8735	0.8745	0.8188	0.8229	0.8124	0.7796	0.8107	0.8754	0.8676
Image 3	4	0.5691	0.5593	0.5605	0.4360	0.5458	0.4990	0.5531	0.5661	0.5598	0.5488
	6	0.7414	0.7377	0.7378	0.6898	0.5048	0.7104	0.7087	0.6402	0.7372	0.6587
	8	0.8280	0.8125	0.8065	0.7647	0.6554	0.7817	0.7246	0.7088	0.8126	0.6813
	10	0.8663	0.8583	0.8507	0.7772	0.8574	0.6977	0.8003	0.5442	0.8477	0.7494
Image 4	4	0.6876	0.6755	0.6755	0.6755	0.6657	0.6434	0.6385	0.6730	0.6746	0.6795
	6	0.7855	0.7810	0.7818	0.7615	0.7281	0.7152	0.7261	0.7646	0.7794	0.782
	8	0.8374	0.8303	0.8233	0.8077	0.7848	0.7912	0.7247	0.7969	0.8199	0.8216
	10	0.8694	0.8562	0.8656	0.8231	0.6977	0.8357	0.8228	0.8438	0.8682	0.8708
Image 5	4	0.7796	0.7695	0.7742	0.7370	0.6891	0.7514	0.7499	0.6413	0.7735	0.7612
	6	0.8573	0.8538	0.8504	0.8320	0.7043	0.8033	0.8265	0.8480	0.8499	0.8447
	8	0.8900	0.8879	0.8900	0.8593	0.8205	0.8512	0.8701	0.8772	0.8887	0.8846
	10	0.9164	0.9134	0.9076	0.8799	0.8323	0.8780	0.8863	0.8753	0.9112	0.9084
Image 6	4	0.7586	0.7404	0.7488	0.7476	0.5164	0.6219	0.7302	0.5437	0.7386	0.7496
	6	0.8252	0.8227	0.8112	0.8159	0.5650	0.7537	0.7364	0.7478	0.8215	0.8145
	8	0.8643	0.8628	0.8577	0.8351	0.4608	0.7692	0.7923	0.7273	0.8645	0.8353
	10	0.8880	0.8829	0.8862	0.7944	0.7184	0.8071	0.8199	0.5512	0.8841	0.8742
Image 7	4	0.6198	0.6198	0.6191	0.5138	0.2837	0.4030	0.3894	0.3804	0.6188	0.6266
	6	0.7519	0.5113	0.5115	0.5782	0.5191	0.4573	0.4652	0.2884	0.7513	0.7895
	8	0.8357	0.5641	0.8300	0.5235	0.5360	0.5801	0.5792	0.3682	0.8283	0.8145
	10	0.8568	0.3171	0.8470	0.4666	0.5364	0.5900	0.5247	0.5319	0.5726	0.8836
Image 8	4	0.7494	0.7559	0.7502	0.6977	0.5501	0.7573	0.7108	0.6869	0.7494	0.7436
	6	0.8242	0.8215	0.8227	0.7983	0.7435	0.7045	0.7708	0.7165	0.8216	0.8256
	8	0.8686	0.5826	0.8682	0.8200	0.7062	0.7879	0.8222	0.8191	0.8669	0.8674
	10	0.8970	0.5995	0.8930	0.8240	0.8085	0.6796	0.8416	0.8397	0.8918	0.8919
Image 9	4	0.7616	0.7470	0.7547	0.7469	0.7440	0.7047	0.7366	0.7555	0.7608	0.75477
	6	0.8312	0.8243	0.8303	0.8041	0.7947	0.7814	0.7996	0.8157	0.8312	0.8164
	8	0.8739	0.8627	0.8736	0.8452	0.6707	0.8326	0.8261	0.8545	0.8676	0.8612
	10	0.8934	0.8909	0.8882	0.8629	0.7996	0.8357	0.8664	0.8682	0.8821	0.8995
Average		0.8090	0.759	0.7970	0.7435	0.6640	0.7175	0.7313	0.6868	0.7959	0.7982

**Table 14:** Comparison of VOI computed by SAMFO-TH, MVO, WOA, FPA, SCA, ACO, PSO, ABC, and MFO with the proposed model using Otsu’s method with N = 4, 6, 8, and 10.

Image	N	VOI									
		SAMFO-TH	MVO	WOA	FPA	SCA	ACO	PSO	ABC	MFO	Proposed
Image 1	4	5.1597	5.1735	5.1635	5.2914	5.1898	5.1906	5.1953	5.5639	5.1738	5.2185
	6	4.6087	4.6091	4.6208	4.7486	4.9652	5.0643	4.8396	4.6954	4.6196	4.5787
	8	4.2244	4.2283	4.2972	4.3304	4.6424	4.7098	4.5490	5.1978	4.2383	4.2931
	10	3.8522	3.8926	0.8799	4.1881	4.9009	4.5518	4.3579	4.5962	3.9094	4.1352
Image 2	4	5.2490	5.2678	5.2622	5.4100	6.0261	5.5241	5.4267	5.6839	5.2694	5.1835
	6	4.6131	4.6186	4.6180	4.7996	5.2373	4.8048	4.9939	5.1233	4.6389	4.4032
	8	4.2176	4.2241	4.2256	4.4096	4.5629	4.6096	4.5286	4.6637	4.2457	4.1839
	10	3.9058	3.9201	3.9192	4.2513	4.2760	4.2297	4.4573	4.2364	3.9104	4.1574
Image 3	4	5.0987	5.1648	5.1315	5.4153	5.2198	5.3013	5.1339	5.1087	5.1476	5.0607
	6	4.4624	4.5006	4.5033	4.6072	5.1735	4.4920	4.5557	4.7943	4.5236	4.355
	8	3.9634	4.0487	4.0924	4.2768	4.6220	4.1960	4.4160	4.4206	4.0406	4.3397
	10	3.6484	3.7003	3.7474	4.1043	3.7382	4.4316	3.9850	4.8480	3.7688	4.0055
Image 4	4	4.9652	5.0588	5.0588	5.0588	5.0924	5.1088	5.1571	5.0646	5.0620	4.81154
	6	4.4858	4.5102	4.4942	4.5252	4.7312	4.7436	4.7174	4.5790	4.5166	4.2371
	8	4.0900	4.1363	4.1466	4.2513	4.4295	4.3004	4.6372	4.2766	4.1893	4.1069
	10	3.7692	3.8670	3.8496	4.1080	4.8438	3.9824	4.0803	3.9572	3.7997	3.6062
Image 5	4	5.1647	5.2732	5.2170	5.3375	5.6024	5.2917	5.2291	5.5993	5.2183	5.1761
	6	4.6169	4.6627	4.6878	4.7562	5.3031	4.9591	4.8554	4.6863	4.7021	4.4915
	8	4.2945	4.3123	4.2890	4.5386	4.7541	4.5276	4.4113	4.3794	4.3081	4.2279
	10	3.9536	3.9790	4.0680	4.2660	4.6134	4.2640	4.2168	4.2744	4.0125	3.8988
Image 6	4	4.9121	5.0424	4.9890	4.9838	5.6897	5.4570	5.0618	5.5668	5.0672	5.1982
	6	4.4774	4.5102	4.5846	4.5189	5.3167	4.7687	4.8922	4.8758	4.4973	5.0782
	8	4.1121	4.1467	4.1851	4.3303	5.6751	4.7612	4.5974	4.7867	4.1232	4.8123
	10	3.8581	3.8961	3.8785	4.4241	5.0560	4.4288	4.3883	5.4037	3.8777	4.0184
Image 7	4	4.0540	4.0541	4.0750	4.3182	4.9103	4.5774	4.6546	4.6716	4.0858	4.0738
	6	3.5037	4.2038	4.1898	3.9669	4.2314	4.3535	4.3375	4.8260	3.5100	3.3603
	8	3.0216	3.9017	3.0608	4.0790	4.0959	3.9632	4.0252	4.5635	3.0545	3.3305
	10	2.7955	4.6133	2.8173	4.2430	4.1140	3.9518	4.0747	4.0944	3.7790	3.1255
Image 8	4	5.2231	5.1586	5.2130	5.3258	5.7569	5.1468	5.3772	5.4878	5.2255	4.6588
	6	4.6964	4.7371	4.7190	4.7938	5.1083	5.2071	4.9315	5.1915	4.7365	4.1033
	8	4.3131	5.2724	4.3166	4.5875	5.0823	4.8286	4.5802	4.6438	4.3328	4.2363
	10	3.9643	5.0740	4.0037	4.4949	4.6658	4.8881	4.4133	4.4484	4.0414	4.0374
Image 9	4	5.2450	5.3662	5.3044	5.3442	5.3740	5.4614	5.3595	5.3094	5.2614	5.1524
	6	4.7590	4.8303	4.7883	4.8910	5.0262	5.0121	4.9621	4.8656	4.7724	4.632
	8	4.3492	4.4895	4.3699	4.5726	5.4184	4.6716	4.7405	4.5477	4.4298	4.3059
	10	4.1460	4.1786	4.1995	4.3524	4.8398	4.5492	4.3874	4.3532	4.2530	4.0671
Average		4.32705	4.51730	4.3046	4.608	4.9523	4.7308	4.6813	4.8162	4.3983	4.3516

**Table 15:** Comparison of MEAN computed by SAMFO-TH, MVO, and WOA using Otsu’s method with N = 4, 6, 8, and 10 for red, green, and blue components.

	N	SAMFO-TH			MVO			WOA		
		R	G	B	R	G	B	R	G	B
Image 1	4	1.6087 × 10^3^	994.5160	845.7528	1.6087 × 10^3^	994.5160	845.7528	1.6087 × 10^3^	994.5160	845.7528
	6	1.6950 × 10^3^	1.0597 × 10^3^	902.2855	1.6955 × 10^3^	1.0599 × 10^3^	902.4505	1.6955 × 10^3^	1.0599 × 10^3^	901.7954
	8	1.7279 × 10^3^	1.0839 × 10^3^	922.7777	1.7283 × 10^3^	1.0844 × 10^3^	922.2913	1.7284 × 10^3^	1.0838 × 10^3^	921.2086
	10	1.7435 × 10^3^	1.0954 × 10^3^	932.5896	1.7443 × 10^3^	1.0961 × 10^3^	933.4105	1.7436 × 10^3^	1.0956 × 10^3^	932.3066
Image 2	4	1.6719 × 10^3^	1.5099 × 10^3^	1.5454 × 10^3^	1.6719 × 10^3^	1.5099 × 10^3^	1.5454 × 10^3^	1.6719 × 10^3^	1.5099 × 10^3^	1.5454 × 10^3^
	6	1.7701 × 10^3^	1.5920 × 10^3^	1.6191 × 10^3^	1.7702 × 10^3^	1.5921 × 10^3^	1.6192 × 10^3^	1.7702 × 10^3^	1.592 l × 10^3^	1.6192 × 10^3^
	8	1.8055 × 10^3^	1.6233 × 10^3^	1.6442× 10^3^	1.8062 × 10^3^	1.6237 × 10^3^	1.6444 × 10^3^	1.8063 × 10^3^	1.6229 × 10^3^	1.6440 × 10^3^
	10	1.8209 × 10^3^	1.6365 × 10^3^	1.6558× 10^3^	1.8216 × 10^3^	1.6374 × 10^3^	1.6566 × 10^3^	1.8218 × 10^3^	1.6374 × 10^3^	1.6556 × 10^3^
Image 3	4	4.2892 × 10^3^	3.0362 × 10^3^	2.5487 × 10^3^	4.2892 × 10^3^	3.0362 × 10^3^	2.5487 × 10^3^	4.2892 × 10^3^	3.0362 × 10^3^	2.5487 × 10^3^
	6	4.4047 × 10^3^	3.1388 × 10^3^	2.6482 × 10^3^	4.4049 × 10^3^	3.1390 × 10^3^	2.6483 × 10^3^	4.4049 × 10^3^	3.1390 × 10^3^	2.6483 × 10^3^
	8	4.4408 × 10^3^	3.1738 × 10^3^	2.6767 × 10^3^	4.441 l × 10^3^	3.1743 × 10^3^	2.6774 × 10^3^	4.441 l × 10^3^	3.1743 × 10^3^	2.6775 × 10^3^
	10	4.4564 × 10^3^	3.1876 × 10^3^	2.6898 × 10^3^	4.4569 × 10^3^	3.1882 × 10^3^	2.6903 × 10^3^	4.4570 × 10^3^	3.1883 × 10^3^	2.6903 × 10^3^
Image 4	4	1.4749 × 10^3^	1.8297 × 10^3^	1.5474 × 10^3^	1.4749 × 10^3^	1.8297 × 10^3^	1.5474 × 10^3^	1.4749 × 10^3^	1.8297 × 10^3^	1.5474 × 10^3^
	6	1.5234 × 10^3^	1.8925 × 10^3^	1.5981 × 10^3^	1.5223 × 10^3^	1.8927 × 10^3^	1.5982 × 10^3^	1.5235 × 10^3^	1.8927 × 10^3^	1.5982 × 10^3^
	8	1.5453 × 10^3^	1.9177 × 10^3^	1.6163 × 10^3^	1.5458 × 10^3^	1.9188 × 10^3^	1.6167 × 10^3^	1.5444 × 10^3^	1.9186 × 10^3^	1.6164 × 10^3^
	10	1.5542 × 10^3^	1.9285 × 10^3^	1.6255 × 10^3^	1.5546 × 10^3^	1.9293 × 10^3^	1.6257 × 10^3^	1.5552 × 10^3^	1.9300 × 10^3^	1.6259 × 10^3^
Image 5	4	3.7536 × 10^3^	3.0007 × 10^3^	3.7919 × 10^3^	3.7536 × 10^3^	3.0007 × 10^3^	3.7919 × 10^3^	3.7536 × 10^3^	3.0007 × 10^3^	3.7919 × 10^3^
	6	3.9152 × 10^3^	3.1253 × 10^3^	3.9104× 10^3^	3.9155 × 10^3^	3.1256 × 10^3^	3.9110 × 10^3^	3.9155 × 10^3^	3.1256 × 10^3^	3.9110 × 10^3^
	8	3.9619 × 10^3^	3.1642 × 10^3^	3.9501 × 10^3^	3.9627 × 10^3^	3.1655 × 10^3^	3.9518 × 10^3^	3.9623 × 10^3^	3.1656 × 10^3^	3.9502 × 10^3^
	10	3.9846 × 10^3^	3.1814 × 10^3^	3.9680 × 10^3^	3.9866 × 10^3^	3.1836 × 10^3^	3.970l × 10^3^	3.986l × 10^3^	3.1836 × 10^3^	3.9678 × 10^3^
Image 6	4	1.8662 × 10^3^	860.0400	601.3654	1.8662 × 10^3^	860.0424	601.3678	1.8662 × 10^3^	860.0424	601.3678
	6	1.9412 × 10^3^	913.4510	634.9386	1.9415 × 10^3^	914.0838	635.1728	1.9415 × 10^3^	914.1091	632.7640
	8	1.9734 × 10^3^	932.0683	648.1786	1.974 l × 10^3^	933.0491	649.1121	1.974l × 10^3^	933.2065	648.1710
	10	1.9874 × 10^3^	941.6650	654.5853	1.9892 × 10^3^	943.0225	655.7298	1.9899 × 10^3^	943.2505	654.2640
Image 7	4	1.3407 × 10^3^	243.8439	40.0194	1.3407 × 10^3^	243.8439	39.9051	1.3407 × 10^3^	243.8395	39.9050
	6	1.3917 × 10^3^	262.7468	43.7509	1.3918 × 10^3^	263.0009	43.7351	1.3918 × 10^3^	262.5177	43.7559
	8	1.4132 × 10^3^	270.2212	45.0938	1.4139 × 10^3^	270.5621	−Inf	1.4136 × 10^3^	270.3070	−Inf
	10	1.4235 × 10^3^	273.0833	48.2657	1.4242 × 10^3^	274.2223	−Inf	1.4244 × 10^3^	274.3484	45.8379
Image 8	4	3.1017 × 10^3^	2.2453 × 10^3^	683.3868	3.1017 × 10^3^	2.2453 × 10^3^	683.3868	3.1017 × 10^3^	2.2453 × 10^3^	683.3868
	6	3.2166 × 10^3^	2.3568 × 10^3^	717.0620	3.2170 × 10^3^	2.3571 × 10^3^	717.3555	3.2170 × 10^3^	2.357 l × 10^3^	717.3477
	8	3.2556 × 10^3^	2.3951 × 10^3^	730.8762	3.2566 × 10^3^	2.3958 × 10^3^	729.9960	3.2567 × 10^3^	2.3959 × 10^3^	730.0061
	10	3.2742 × 10^3^	2.4123 × 10^3^	735.7058	3.276l × 10^3^	2.4129 × 10^3^	−Inf	3.2760 × 10^3^	2.4136 × 10^3^	−Inf
Image 9	4	1.3756 × 10^3^	1.9760 × 10^3^	1.7498 × 10^3^	1.3756 × 10^3^	1.9760 × 10^3^	1.7498 × 10^3^	1.3732 × 10^3^	1.9760 × 10^3^	1.7498 × 10^3^
	6	1.4472 × 10^3^	2.0578 × 10^3^	1.8340 × 10^3^	1.4473 × 10^3^	2.0566 × 10^3^	1.8344 × 10^3^	1.4468 × 10^3^	2.0532 × 10^3^	1.8314 × 10^3^
	8	1.4808 × 10^3^	2.0862 × 10^3^	1.8657 × 10^3^	1.4813 × 10^3^	2.0870 × 10^3^	1.8652 × 10^3^	1.4799 × 10^3^	2.0874 × 10^3^	1.8642 × 10^3^
	10	1.4947 × 10^3^	2.1044 × 10^3^	1.8806 × 10^3^	1.4954 × 10^3^	2.1055 × 10^3^	1.8806 × 10^3^	1.496 l × 10^3^	2.1060 × 10^3^	1.8791 × 10^3^
Average		1.71 × 10^3^	1.44 × 10^3^	6.15 × 10^2^	1.58 × 10^3^	1.66 × 10^3^	1.06 × 10^3^	1.73 × 10^3^	1.47 × 10^3^	1.06 × 10^3^

**Table 16:** Comparison of MEAN computed by SAMFO-TH, MVO, and WOA using Kapur’s method with N = 4, 6, 8, and 10 for red, green, and blue components.

Image	N	SAMFO-TH			MVO			WOA		
		R	G	B	R	G	B	R	G	B
Image 1	4	18.6011	18.3224	18.1192	18.6013	18.3228	18.1249	18.6013	18.3228	18.1259
	6	23.7425	23.4112	23.3245	23.7519	23.4374	23.3455	23.7535	23.4374	23.3129
	8	28.4075	27.9424	27.9925	28.3981	27.9889	28.009	28.3251	27.9161	28.0052
	10	32.5752	31.9895	32.2486	32.5213	32.0989	32.3002	32.4842	32.0061	32.3188
Image 2	4	17.5802	17.5424	18.5899	17.5805	17.5505	18.5933	17.5805	17.5505	18.5933
	6	22.5676	22.5665	23.8485	22.5912	22.5931	23.8607	22.5974	22.5936	23.858
	8	27.1253	27.0319	28.7393	27.0672	27.0603	28.7825	27.1326	27.0716	28.8099
	10	31.2837	31.1974	33.0202	31.2257	31.3003	33.0647	31.2572	31.3245	33.1572
Image 3	4	17.6776	17.5996	17.3878	17.6777	17.6	17.3886	17.6777	17.6003	17.3885
	6	22.9482	22.6386	22.5821	22.9479	22.6474	22.6717	22.9421	22.6488	22.6716
	8	27.7786	27.2642	27.4267	27.7695	27.3503	27.4994	27.7393	27.3631	27.5042
	10	32.2057	31.542	31.7474	32.1717	31.6993	31.883	32.0405	31.6441	31.9212
Image 4	4	17.5082	17.9799	17.4413	17.5028	17.9983	17.4419	17.5079	17.9982	17.4415
	6	22.4998	23.0284	22.2581	22.5099	23.035	22.282	22.4832	23.0501	22.282
	8	26.9272	27.5976	26.7533	26.9801	27.6366	26.8219	26.8891	27.6498	26.7813
	10	30.8946	31.7548	30.7424	30.8572	31.9019	30.6887	30.8505	31.8949	30.8114
Image 5	4	18.053	17.5873	17.6037	18.0534	17.5877	17.6043	18.0534	17.5878	17.6043
	6	23.0011	22.3708	22.7447	23.0067	22.3848	22.7505	23.0076	22.3856	22.7519
	8	27.5962	26.6633	27.3121	27.5879	26.726	27.3687	27.5815	26.7322	27.3685
	10	31.8751	30.5385	31.4943	31.8596	30.5861	31.5435	31.8028	30.5624	31.5951
Image 6	4	18.372	17.6817	16.5909	18.3723	17.6815	16.591	18.3724	17.6718	16.5849
	6	23.7047	22.9559	21.4418	23.707	22.9787	21.4523	23.6896	22.9848	21.4489
	8	28.4616	27.5295	25.6948	28.4571	27.6226	25.5179	28.4069	27.6628	25.6499
	10	32.5313	31.6233	29.2761	32.6353	31.6326	28.9661	32.4703	31.6589	29.3176
Image 7	4	17.9999	16.0346	12.1942	17.9919	16.0339	12.0486	17.9972	16.0369	11.7758
	6	23.235	20.7147	15.5653	23.2065	20.7138	14.0047	23.1991	20.487	14.4237
	8	27.9628	24.821	17.944	27.9217	24.5978	16.2308	27.9	24.6795	16.8152
	10	32.0804	28.3688	19.5	31.9972	27.5907	17.9641	32.0429	27.9357	18.1042
Image 8	4	18.5996	18.6285	15.9913	18.5996	18.6286	15.9914	18.5996	18.6287	15.9912
	6	23.9028	23.8893	20.3696	23.9016	23.8302	20.3211	23.8386	23.8394	20.1171
	8	28.623	28.5837	24.2063	28.6191	28.4635	23.7059	28.5621	28.576	23.7238
	10	32.9212	32.8709	27.55	32.8936	32.727	26.2619	32.785	32.9277	26.9726
Image 9	4	17.8777	17.8204	18.2427	17.8772	17.821	18.2429	17.8778	17.8209	18.2428
	6	22.7729	22.6426	23.5334	22.6822	22.657	23.5311	22.685	22.6579	23.5242
	8	27.1134	26.9126	28.2759	27.0729	26.803	28.2644	27.1241	26.6663	28.1919
	10	31.3765	30.6957	32.4466	31.3039	30.5338	32.4963	31.3781	30.5634	32.4157
Average		24.89989	24.12585	22.79991	24.86062	23.99844	22.47571	24.82708	24.03807	22.54371

**Table 17:** Average MEAN of fitness with Kapur’s and Otsu’s methods on optimization techniques for MVO, WOA, PFA, SCA, ACO, PSO, ABC, MFO, and SAMFO-TH and on the proposed approach for nine images considered with N = 4, 6, 8, and 10.

	MEAN with KAPUR’s Method			MEAN with OTSU’s Method		
Methods	R	G	B	R	G	B
Proposed	42.7288	41.8091	41.9909	1.83 × 10^11^	2.59 × 10^11^	2.71 × 10^11^
MVO	24.8606	23.9984	22.4757	1.58 × 10^3^	1.66 × 10^3^	1.06 × 10^3^
WOA	24.8270	24.0380	22.5437	1.73 × 10^3^	1.47 × 10^3^	1.06 × 10^3^
PFA	24.6788	24.1296	22.8015	7.10 × 10^2^	5.69 × 10^2^	4.04 × 10^2^
SCA	22.6041	21.2926	19.9449	9.95 × 10^2^	2.09 × 10^3^	1.89 × 10^3^
ACO	28.8877	27.5038	25.4009	1.02 × 10^3^	1.38 × 10^3^	2.31 × 10^3^
PSO	23.5873	22.5467	20.8294	8.92 × 10^2^	1.12 × 10^3^	1.25 × 10^3^
ABC	24.2798	23.3019	21.5508	1.47 × 10^3^	2.04 × 10^3^	1.86 × 10^3^
MFO	23.7660	22.7604	21.2234	1.47 × 10^3^	2.04 × 10^3^	1.86 × 10^3^
SAMFO-TH	24.8998	24.1258	22.7999	1.71 × 10^3^	1.44 × 10^3^	6.15 × 10^2^

**Table 18:** Steps for implementation of the Proposed method on a color image.

Step	Operation
1:	Read a color image I and separate it into I_R_, I_G_, and I_B._ For RGB image c = 1,2,3 and for gray image c = 1.
2:	Obtain energy curves for RGB images E^R^, E^G^, and E^B^.
3:	Calculate the probability distribution using Equation (3) and the histograms.
4:	Initialize the parameters: ${Iter}_{max}$, ${Iter}_{local},$ δ, and N
5:	Initialize a population $S_{t}^{c}$ of N random particles with k dimensions.
6:	Find $w_{i}^{c}$ and $\mu_{i}^{c}$; evaluate $S_{t}^{c}$ in the objective function $f_{otsu}$ or $f_{Kapur}$ depends on the thresholding method to find threshold values for segmentation.
7:	Compute the charge of each particle using Equation (18), and with Equations (19) and (20) compute the total force vector.
8:	Move the entire population $S_{t}^{c}$ along the total force vector using Equation (21).
9:	Apply the local search to the moved population and select the best elements of this search depending on their values of the objective function.
10:	The t index is increased in 1. If t ≥ ${Iter}_{max}$ or if the stop criteria are satisfied the algorithm finishes the iteration process and jumps to step 11. Otherwise, jump to step 7.
11:	Select the particle that has the best $x_{i}^{B}$ objective function value using $f_{otsu}$ or $f_{Kapur}$ from Equation (9)or Equation(14).
12:	Apply the best thresholds values contained in $x_{i}^{B}$ to the input image I as per Equation (2).

## Data Availability

The data set used for experimentation in this study is taken from BSDS500 and USC-SIPI, and this data is available in the public domain.
